# Evidence-based clinical practice guidelines for inflammatory bowel disease

**DOI:** 10.1007/s00535-018-1439-1

**Published:** 2018-02-10

**Authors:** Katsuyoshi Matsuoka, Taku Kobayashi, Fumiaki Ueno, Toshiyuki Matsui, Fumihito Hirai, Nagamu Inoue, Jun Kato, Kenji Kobayashi, Kiyonori Kobayashi, Kazutaka Koganei, Reiko Kunisaki, Satoshi Motoya, Masakazu Nagahori, Hiroshi Nakase, Fumio Omata, Masayuki Saruta, Toshiaki Watanabe, Toshiaki Tanaka, Takanori Kanai, Yoshinori Noguchi, Ken-ichi Takahashi, Kenji Watanabe, Toshifumi Hibi, Yasuo Suzuki, Mamoru Watanabe, Kentaro Sugano, Tooru Shimosegawa

**Affiliations:** 1 Guidelines Committee for Creating and Evaluating the ‘‘Evidence-Based Clinical Practice Guidelines for Inflammatory Bowel Disease in Japan’’, The Japanese Society of Gastroenterology (JSGE), 6F Shimbashi i-MARK Building, 2-6-2 Shimbashi, Minato-ku, Tokyo, 105-0004 Japan; 2 Ofuna Central Hospital, 6-2-24 Ofuna, Kamakura-shi, Kanagawa 247-0056 Japan

**Keywords:** Inflammatory bowel disease, Ulcerative colitis, Crohn’s disease, Guidelines, Evidence, Consensus

## Abstract

Inflammatory bowel disease (IBD) is a chronic disorder involving mainly the intestinal tract, but possibly other gastrointestinal and extraintestinal organs. Although etiology is still uncertain, recent knowledge in pathogenesis has accumulated, and novel diagnostic and therapeutic modalities have become available for clinical use. Therefore, the previous guidelines were urged to be updated. In 2016, the Japanese Society of Gastroenterology revised the previous versions of evidence-based clinical practice guidelines for ulcerative colitis (UC) and Crohn’s disease (CD) in Japanese. A total of 59 clinical questions for 9 categories (1. clinical features of IBD; 2. diagnosis; 3. general consideration in treatment; 4. therapeutic interventions for IBD; 5. treatment of UC; 6. treatment of CD; 7. extraintestinal complications; 8. cancer surveillance; 9. IBD in special situation) were selected, and a literature search was performed for the clinical questions with use of the MEDLINE, Cochrane, and Igaku Chuo Zasshi databases. The guidelines were developed with the basic concept of the Grading of Recommendations Assessment, Development, and Evaluation (GRADE) system. Recommendations were made using Delphi rounds. This English version was produced and edited based on the existing updated guidelines in Japanese.

## Introduction

1. Purpose of the revised guidelines

The purpose of these clinical practice guidelines is to improve the patient outcome by providing appropriate clinical indices to health care providers involved in the management of IBD.

2. Revision process

1) Basic principle

We adopted the basic concept of GRADE system whenever possible. Clinical indices were based on the summary of evidence by systematic review, and recommendation grades were determined by the consensus of members, not necessarily correlated with level of evidence.

2) Methods for revision

Clinical questions (CQ) were completely reexamined. For 9 categories (1. clinical features of IBD; 2. diagnosis; 3. general consideration in treatment; 4. therapeutic interventions for IBD; 5. treatment of UC; 6. treatment of CD; 7. extraintestinal complications; 8. cancer surveillance; 9. IBD in special situation), a total of 59 CQs were extracted. The availability of health insurance coverage is described in the commentary, as indicated.

These recommendations are evidence based as much as possible. The working group formally reviewed and analyzed the recently published literatures relevant to the clinical practice of IBD (MEDLINE, Cochrane review for English literatures and Igaku Chuo Zasshi for Japanese literatures) systematically searched from 1983 up to June 2012, as well as additional literatures searched by individual members of the group up to 2015. Determination of the level of evidence was in accordance with the method of GRADE system. Initially set to 4 levels of evidence (high/moderate/low/very low) according to study design, necessary downward reset was made after consideration on risk of bias by examining the study method.When the evidence was neither consistent nor available, the statements were made based on the opinion of the members of the group. The grade of recommendation was applied only to the statements concerning the clinical interventions.

The appropriateness of each statement was determined by voting of 13 committee members with 9 stage (9 = most appropriate, 1 = most inappropriate) scoring. After Delphi rounds, for those statements with median score 9 or 8, strong recommendation was given (recommended), and weak recommendation (suggested) for median score of 7. Recommendation used for GRADE system was adopted with minor modification.

**Table:** 

Grade of recommendation	Criteria (mean Delphi Score)	Interpretation
1: Strong recommendation	8–9	Recommend to do Recommend not to do
2: Weak recommendation	7	Suggest to do Suggest not to do

The draft was submitted to the assessment committee draft to the evaluation committee, collected evaluation comments, fed back to the committee members in charge, and necessary modifications made. This process was repeated once more, and the final plan was formulated.

3. Internal review

The statements and comments received the public comments by members of the Japanese Gastroenterology, and members of the Research Group of Intractable Inflammatory Bowel Disease subsidized by the Ministry of Health, Labour and Welfare of Japan, reviewed for the appropriateness. The final proposal was made after minor revision.

4. Application of guidelines

These guidelines are intended for use of clinical practitioners in various settings in the care of patients with IBD. Recommendations are standard or preferred approaches, but should be used flexibly for individual patients. Clinical Practice Guidelines Committee of the Japanese Society of Gastroenterology is responsible for the description contents, but the responsibility for outcomes in practice should be attributed to individual clinical practitioners. In addition, the contents of these guidelines should not be used as a legal basis such as medical lawsuits.

5. Future issues

With the accumulation of new evidence and the approval of new diagnostic and therapeutic agents, strategies for the management of IBD will change considerably over the next few years. The current guidelines are subject to revision in 4–5 years.

## 1. Clinical features of inflammatory bowel disease

**CQ1-01. Definitions and pathophysiology of inflammatory bowel disease**

### Statements

- Inflammatory bowel disease (IBD) refers to diseases of chronic or remitting/relapsing intestinal inflammation and includes primarily ulcerative colitis (UC) and Crohn’s disease (CD).
- UC is a diffuse non-specific inflammatory disease of unknown cause that continuously affects the colonic mucosa proximal from the rectum and often forms erosions and/or ulcers.
- CD is a chronic inflammatory disease of unknown cause, characterized by discontinuously affected areas with transmural granulomatous inflammation and/or fistula. CD can affect any region in the digestive tract from the mouth to the anus, but is more likely to involve the small and large intestines (especially the ileocecum) and the perianal region.

### Comments

IBD refers to diseases of a chronic or remitting/relapsing intestinal inflammation. This guideline describes UC and CD as the major forms of inflammatory bowel diseases of unknown etiology. Both diseases develop complicated pathology with unknown causes and mainly affect the gastrointestinal tract, resulting in various clinical symptoms.

UC is a diffuse non-specific inflammatory disease of unknown cause that continuously affects the colonic mucosa proximal from the rectum and often forms erosions and/or ulcers. It frequently repeats cycles of relapse and remission during its course and may be accompanied by extraintestinal complications. When it extensively affects the large intestine for a long period of time, a risk of developing cancer increases [1].

CD is a chronic inflammatory disease of unknown cause, characterized by discontinuously affected areas with transmural granulomatous inflammation and/or fistula. It can affect any region in the digestive tract from the mouth to the anus, but is more likely to involve the small and large intestines (especially the ileocecum) and the perianal region [2].

The cause of IBD has yet to be identified, but it is considered to develop as the result of abnormal intestinal immunity and altered gut microbiota caused by environmental factors such as diet and infection in genetically susceptible individuals. Patients with IBD often experience impaired daily quality of life (QOL) since both diseases develop in young ages, present symptoms such as abdominal pain, diarrhea, and bloody stool, and chronical progression with repeated cycles of relapse and remission. In addition, it may develop extraintestinal complications in systemic organs such as the joints, the skin, and the eyes.

The incidence of colorectal cancer (CRC) is significantly increased in UC patients who have extensive lesions for a long period of time, and it is also known that the incidence of cancers in the small and large intestines, especially in the rectum and anal canal region, is high in CD patients. Therefore, an efficient surveillance strategy for cancer development is expected to be established. IBD is not considered to be a disease that significantly affects the patients’ life prognosis, although IBD patients have slightly shorter life prognosis compared to normal individuals.

UC and CD are collectively referred to as IBD because the two diseases share common or similar features; however, disease location, morphology, and pathophysiology are clearly different between them, and they are considered to be independent diseases. Moreover, it is necessary to classify them because diagnostic procedures, therapeutic interventions, and follow-up observation are somewhat different. Notably, it is called “IBD unclassified” when colonic lesions have the features of IBD which cannot be classified as UC or CD.

**CQ1-02. What is the epidemiology of IBD?**

### Statements

- The number of IBD patients has been increasing year by year; it is estimated that there are over 160,000 patients with UC (approximately 100 per 100,000) and about 40,000 patients with CD (approximately 27 per 100,000).
- Both UC and CD develop at relatively young ages; patients are more likely to develop the disease between their late 10s and early 30s.
- In Western countries, there tend to be more women among IBD patients, especially CD patients, but there is a male predominance in Japan.
- The cause of IBD has not yet been clarified; however, it is considered that inflammation occurs in genetically predisposed individuals as the result of impairment of the regulatory mechanisms of the intestinal mucosal immune system, which is caused by the involvement of various environmental factors.

### Comments

In Japan, the number of IBD patients has been increasing year by year; it is estimated by the issued numbers of certificates of recipients of medical service and certificates of registration in 2013 that there are over 160,000 patients with UC (approximately 100 per 100,000) and about 40,000 patients with CD (approximately 27 per 100,000) [3]. However, the recent accurate incidence and prevalence of IBD are unknown because a nationwide epidemiological survey has not been conducted since 1991.

Both UC and CD develop at relatively young ages; patients are more likely to develop the disease between their late 10s and early 30s. Nevertheless, patients’ ages gradually shift to an elderly population and opportunities to see elderly patients with IBD are also increasing in these days because elderly onset IBD is not rare, IBD patients have a relatively good life prognosis and a long disease course, and the general population of elderly has been recently increasing.

The incidence and prevalence of IBD in Western countries are different among regions, but higher in most countries compared to Japan; and there tend to be more women in IBD patients in Western countries, especially CD patients; on the other hand, there is a male predominance in Japan.

The cause of IBD has not yet been clarified; however, there is an international consensus that inflammation occurs in genetically predisposed individuals as the result of impairment of the regulatory mechanisms of the intestinal mucosal immune system, which is caused by the involvement of various environmental factors. A certain degree of genetic influence is suggested by the slightly higher prevalence of IBD in blood-relations and reports of intra-familial accumulation of IBD. Research on disease susceptibility genes is underway also in Japan, but results consistent with Western countries have not been obtained partly because susceptibility genes of Japanese patients are different from foreign countries.

**CQ1-03. What are the cause of and risk factors for UC?**

### Statements

- Several loci are reported to be associated with UC (Evidence level: C).
- The cause of UC has yet to be identified, but certain kinds of food composition are reported to be associated with UC (Evidence level: C).
- It has been reported that smoking is protective against UC, but its causality has not been clear (Evidence level: C).
- The use of oral contraceptives is associated with the development of UC; the use of nonsteroidal anti-inflammatory drugs (NSAIDs) is associated with the development and worsening of IBD (Evidence level: C).

### Comments

According to the meta-analysis of 15 genome-wide association studies including about 10,000 patients of UC, 163 loci are associated with IBD; of these, 133 loci are associated with UC [4]. A Japanese multicenter case–control study showed that an intake of sugar candies is associated with the development of UC; in addition, consumption of vitamin C provides a negative association in the development of UC [5]. Moreover, a meta-analysis examining the association between smoking and UC suggested that current smokers provided a negative association in the development of UC (odds ratio (OR) 0.58, 95% confidence interval (CI) 0.45–0.75), on the other hand, history of tobacco use was a risk factor for UC (OR 1.79, 95% CI 1.37–2.34) [6]. Therefore, the causal association between smoking and UC remains undetermined. In addition, it is also necessary to consider that smoking is a risk factor for other diseases than UC. A meta-analysis in 2000 demonstrated that the OR of appendectomy for the development of UC was 0.31 (95% CI 0.25–0.38) [7]. A meta-analysis in 2008 reported that the relative risk of oral contraceptives for the development of UC was 1.53 (95% CI 1.21–1.94) [8]. Additionally, the case–control study including 60 patients with IBD (24 patients of UC, 36 patients of CD) in the United States (US) showed that the OR of NSAIDs for worsening or new development of IBD was 20.3 (95% CI 2.6–159.7) [9].

**CQ1-04. What are the cause of and risk factors for CD?**

### Statements

- Several loci are reported to be associated with CD (Evidence level: C).
- The cause of CD has yet to be identified, but certain kinds of food composition are considered to be associated with the cause of CD (Evidence level: C).
- Smoking is a risk factor for CD (Evidence level: C).
- The use of oral contraceptives is associated with the development of CD; the use of NSAIDs is associated with the worsening and development of IBD (Evidence level: C).

### Comments

According to the meta-analysis of genome-wide association studies with inclusion of about 15,000 CD patients, it has been reported that 140 out of 163 loci linked to IBD are associated with CD [4]. A multicenter case–control study from Japan showed that an intake of fat, sugar candies, sugar, sweetener, unsaturated fatty acid, and vitamin E were associated with the development of CD [5]. A meta-analysis on the association between smoking and CD suggested that the OR of current smokers and past smokers was 1.76 (95% CI 1.4–2.22) and 1.30 (95% CI 0.97–1.76), respectively [6]. A meta-analysis in 2008 demonstrated that the relative risk for CD after appendectomy is 1.61 (95% CI 1.28–2.02), but the validity of the study is questionable due to the substantial heterogeneity among the studies included in the meta-analysis [10]. The meta-analysis on oral contraceptives in 2008 demonstrated that the relative risk for CD during the use of oral contraceptives was 1.51 (95% CI 1.17–1.96) [8]. Additionally, the case–control study including 60 patients of IBD (24 patients of UC, 36 patients of CD) in the US showed that the OR of NSAIDs for worsening and development of IBD was 20.3 (95% CI 2.6–159.7) [9].

**CQ1-05. How should the stage, classification, and severity of IBD be evaluated?**

### Statements

- Selection of treatment for UC varies depending on the stage, extent, and severity of the disease. In CD, it is essential to determine the location, pattern, activity, and severity of the disease.
- UC can be divided into two stages: (1) the active stage characterized by the presence of symptoms and endoscopically active mucosal lesions; (2) the remission stage characterized by resolution of symptoms and disappearance of the endoscopically active mucosal findings.
- Depending on the disease extent, UC can be divided into proctitis, distal colitis (up to the sigmoid colon), left-sided colitis (up to the splenic flexure), and pancolitis.
- The severity of UC can be classified into mild, moderate, and severe, based on clinical symptoms and signs, and blood tests (Table 1).

**Table 1:** Classification of severity of ulcerative colitis

	Severe	Moderate	Mild
(1) Bowel movements (no. per day)	≧ 6	Between mild and severe	≦ 4
(2) Blood in stools	(+++)		(+) ~ (−)
(3) Pyrexia	≧ 37.5 °C		No
(4) Pulse	≧ 90/min		No
(5) Anemia	Hb ≦ 10 g/dl		No
(6) ESR^a^	≧ 30 mm/h		Normal

Patients are classified as severe if they present both (1) and (2) plus at least one of (3) or (4), while satisfying 4 or more out of 6 features. Patients with extremely severe symptoms are classified as fulminant, and further divided into acute fulminant or relapsing fulminant types. Diagnostic criteria of fulminant colitis: all of the below

① Satisfy criteria of severe cases

② Bloody diarrhea 15 or more per day continuously

③ Persistent high fever ≧ 38.0 °C

④ White blood cell count ≧ 10,000/mm^3^

⑤ Severe abdominal pain

^a^Erythrocyte sedimentation rate
- Since inflammation of CD tends to develop in the small and large intestines (especially the ileocecum), and the perianal region, CD is divided into the ileal-type, colonic-type, and ileocolonic-type.
- The disease patterns of CD are divided into three types: (1) non-stricturing non-penetrating type, (2) penetrating type, and (3) stricturing type.
- The Crohn’s disease activity index (CDAI), the International Organization for the study of IBD (IOIBD) index, and the Harvey–Bradshaw index have been proposed as indicators of CD activity, but neither of them is commonly used in clinical practice.

### Comments

The pathophysiology of IBD is complicated; therefore, it is essential to accurately determine the disease condition to appropriately treat the disease. Selection of the treatment of UC varies depending on the stage, extent, and severity of the disease. In CD, it is essential to determine the location, pattern, activity, and severity of the disease.

It is common to divide the stage of UC into “the active stage”, in which patients complain of bloody stools and endoscopy reveals loss of vascular pattern, friable mucosa, and erosions and/or ulcers, and “the remission stage”, in which bloody stools resolve and the endoscopic findings of the active disease disappear, and vascular pattern reappears. In addition, UC can be divided into the following types depending on the extent of lesions: proctitis, distal colitis (up to the sigmoid colon), left-sided colitis (up to the splenic flexure), and pancolitis. Since “pancolitis” may cause a misunderstanding that the entire colon is affected, a disease with lesions beyond the splenic flexure may be referred to as “extensive colitis”. The severity of UC is often classified by the definitions developed by the Research Group for Intractable Inflammatory Bowel Disease (Table 1); the severity is graded as “mild” when (1) the frequency of defecation is 4 times/day or less; (2) bloody stools are slight if exist, and (3) systemic symptoms such as fever, palpitation, and anemia are absent; “severe” when (1) the frequency of defecation is 6 times/day or more; (2) apparent bloody stool is present, and (3) systemic symptoms such as fever, palpitation, and anemia are present; “moderate” when the clinical features are in-between “mild” and “severe” [11–14].

Inflammation of CD tends to develop in the small and large intestines (especially the ileocecum), and the perianal region, and CD is divided into the ileal-type, colonic-type, and ileocolonic-type. Since CD may develop not only the gastrointestinal lesions but also extraintestinal manifestations, it is necessary to evaluate their systemic effects. Treatment plans can vary depending on the affected regions. There is an international consensus that the disease behavior of CD is divided into three types: (1) non-stricturing non-penetrating type, (2) penetrating type, and (3) stricturing type. It is important to determine the disease behavior in order to choose appropriate treatments [15].

Moreover, it is also necessary to determine the activity of the disease. This is because treatments are different between the remission stage, where patients have mild or no symptoms, and the active stage, where various symptoms may affect patients’ QOL. The CDAI was developed to measure the activity of CD in clinical trials [16], and its validity has been verified; however, it is cumbersome to use in the daily clinical setting. The IOIBD index is a simple indicator which can be used to distinguish between the active stage and the remission stage, but it does not mean that treatments of CD can be chosen based on this index itself. It is confirmed that the Harvey–Bradshaw index which uses only clinical indicators has a relatively favorable correlation with the CDAI [17]. In general clinical practice, severity is often comprehensively determined by patients’ subjective symptoms, clinical and laboratory findings, etc.; however, the Research Group for Intractable Inflammatory Bowel Disease advocates the severity evaluation criteria that incorporate the CDAI and other indicators (Table 2) [18].

**Table 2:** Classification of severity of Crohn’s disease

	CDAI	Complication	Inflammation (CRP)	Response to treatment
Mild	150–220	No	Slightly elevated	
Moderate	220–450	No clinically significant complications (e.g., bowel obstruction)	Significantly elevated	Not responding to treatments for mild cases
Severe	450<	Significant complication (e.g., bowel obstruction, abscess formation)	Extremely elevated	Refractory

Severity should be graded based on the factors shown above in treating patients

## 2. Diagnosis (Figs. 1 and 2)

**CQ2-01. How should IBD be diagnosed?**

**Fig. 1:**
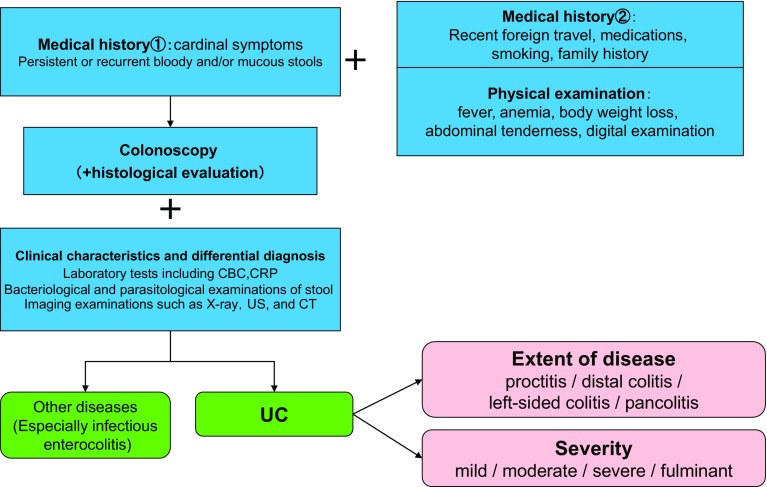
Diagnostic approach of UC

**Fig. 2:**
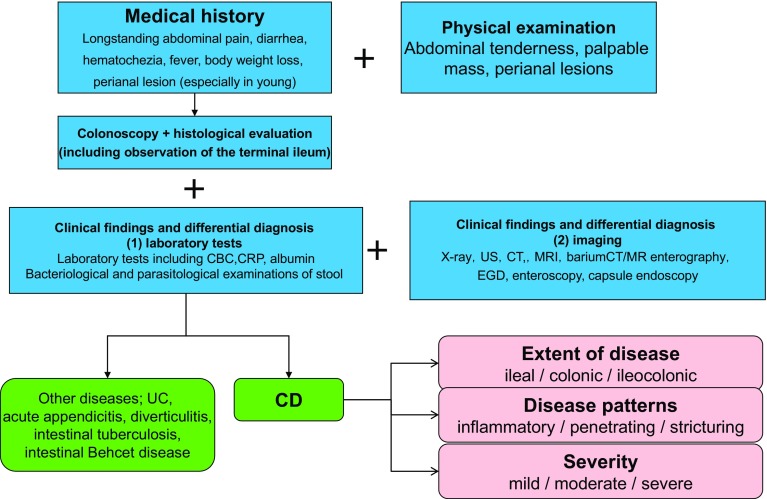
Diagnostic approach of CD

### Statements

- A diagnosis of IBD is suspected by medical history, and characteristic findings of physical examinations, and established by typical findings of imaging examinations such as endoscopy.
- It is often necessary to differentiate infectious enterocolitis from UC.
- The symptoms of the acute stage of CD may resemble those of acute appendicitis or colonic diverticulitis, and it may be difficult to differentiate intestinal tuberculosis or intestinal Behçet’s disease from CD before the final diagnosis of CD is established.
- Once a diagnosis of IBD has been established, the activity, severity, and disease extent should be evaluated to provide appropriate therapeutic interventions.

### Comments

The diagnosis of IBD is usually established by characteristic clinical findings and typical findings of imaging examinations such as endoscopy. However, since there are a number of diseases that are difficult to distinguish even with highly accurate imaging techniques, it is important to first narrow down the differential diagnosis by the appropriate medical history taking and physical examination to obtain an accurate diagnosis and to establish an efficient diagnostic strategy.

The first step in establishing a diagnosis is to suspect IBD. When a patient presents with abdominal symptoms such as repeated abdominal pain or diarrhea, IBD should be included in one of the differential diagnosis regardless of age. It is often necessary to distinguish UC from infectious enterocolitis, especially *Campylobacter*, enteroinvasive *Escherichia coli*, and amoebic dysentery. Excluding these diseases by bacteriological and parasitological examinations is indispensable to make a diagnosis of UC. The symptoms of the acute stage of CD may resemble those of acute appendicitis or colonic diverticulitis, and it may be difficult to differentiate intestinal tuberculosis or intestinal Behçet’s disease from CD before the final diagnosis of CD is established.

Once a diagnosis of IBD has been established, the activity, severity, and disease extent should be evaluated to provide the appropriate therapeutic interventions.

Endoscopy and various imaging techniques are useful for determining the extent and severity of the disease. Furthermore, clinical evaluation for intestinal and extraintestinal complications is also necessary [1, 2].

Refer to CQ2-06 for the diagnostic criteria for UC and CQ2-07 for CD.

**CQ2-02. What are symptoms/physical findings that should raise suspicion of IBD?**

### Statements

- A diagnosis of UC should be suspected when a patient, especially young, presents with persistent or recurrent bloody diarrhea accompanied with abdominal pain or frequent bowel movements.
- A diagnosis of CD should be suspected when a patient, especially young, presents with chronic abdominal pain and diarrhea accompanied with bloody stools, weight loss, fever, or perianal lesions.
- Physical findings of UC are not specific, but abdominal tenderness that reflects the extent and/or severity of the disease, and mucous and bloody stools on a digital rectal examination are often seen.
- In CD, physical examinations may reveal tenderness or a palpable mass that coincides with the affected areas, and signs of bowel obstruction may be observed. Patients may be diagnosed with the perianal lesions.

### Comments

The cardinal symptom of UC is bloody diarrhea, sometimes accompanied with abdominal pain or frequent bowel movements. Therefore, if a patient has persistent or recurrent bloody and/or mucous stools or the history of those symptoms, UC is suspected. It is necessary to take a history of recent foreign travel, medications (especially antimicrobials), and family history. In mild patients, physical examinations often reveal no abnormal findings, but patients with more severe disease demonstrate fever, anemia, weight loss, abdominal tenderness, and fresh blood on a digital rectal examination [1].

The cardinal symptoms of CD are similar to UC. Patients frequently present with chronic abdominal pain and/or diarrhea, with fewer occurrences of bloody stools compared to UC, and mucous and bloody stools seen in UC are rare. In addition, there is a higher possibility that patients demonstrate weight loss, fever, and perianal lesions than UC. Physical examinations may reveal tenderness and a palpable mass in the area affected by CD, and signs of bowel obstruction may be observed. Patients may often be diagnosed with the perianal lesion [2].

**CQ2-03. What are the useful laboratory examinations for the diagnosis of IBD?**

### Statements

- On a complete blood cell count, the presence and severity of anemia are evaluated, and the severity of inflammation can be presumed by leukocyte count (and differentiation) and platelet count.
- Inflammatory markers (CRP, erythrocyte sedimentation rate) are correlated with the activity of the disease.
- Albumin is not only an index of nutrition, but also an index of severity of IBD.
- Infectious enterocolitis should be excluded by bacteriological and parasitological examinations.
- Fecal calprotectin is being introduced as a marker of activity of inflammation.

### Comments

When IBD is suspected on clinical findings, laboratory tests of blood and stool, in parallel with imaging investigations, should be conducted. A complete blood cell count is a useful indicator for evaluating the severity of IBD. It can evaluate the presence and severity of anemia, and presume the severity of inflammation by leukocyte count (and differentiation) and platelet count. Inflammatory markers (CRP, erythrocyte sedimentation rate) are correlated with the activity of the disease; however, it should be reminded that normalized values do not necessarily suggest disappearance of inflammation. A decreased albumin level is not only an index of nutrition, but also an index of severity of IBD. Infectious enterocolitis should be excluded by bacteriological and parasitological examinations. Fecal immunological occult blood test (FIT) can be used for monitoring activity of inflammation or assessing of mucosal healing, but its usefulness has not been sufficiently validated [19].

Besides conventional inflammatory markers such as CRP and erythrocyte sedimentation rate, in the near future, fecal calprotectin will be introduced as a marker to assess the activity of inflammation and applied for clinical use [20].

***1) Endoscopy (including biopsy sampling)***

**CQ2-04. How should endoscopy be used for the diagnosis of UC?**

### Statements

- It is recommended that colonoscopy should be conducted to establish a definite diagnosis of UC when it is suspected based on clinical findings (Recommendation grade: 1 (9), Evidence level: D).
- Colonoscopy is recommended not only for establishing a definite diagnosis, but also for determining the disease severity and response to treatment, and for surveillance of cancer development (Recommendation grade: 1 (9), Evidence level: D).

### Comments

Colonoscopy should be conducted to establish a diagnosis of UC when it is suspected based on clinical findings [1, 21–23]. The indications of colonoscopy in UC patients include the establishment of the definite diagnosis, assessment of disease severity, judgment of response to treatment, and surveillance of cancer development. Histological examination of biopsy should be done if deemed necessary. Particularly in patients who are diagnosed with UC for the first time, total colonoscopy should be performed, if possible, in order to determine the appearance, severity, and, extent of colonic lesions, excluding other diseases. However, it is not necessary to perform total colonoscopy early in patients with clinically severe activity because the disease may be worsened by the endoscopic procedure itself or preparation for colonoscopy. Oral bowel cleansing is used as a bowel preparation, but it is often possible to perform colonoscopy without a bowel preparation in patients who have frequent diarrhea and bloody stools in the active stage of UC.

Typical endoscopic findings of UC include loss of vascular pattern, granular and easily bleeding mucosa, and ulceration; those findings are observed in a continuous manner [14]. The mucosa is diffusely affected, loses the normal vascular patterns, and presents a coarse or microgranular appearance. Furthermore, the mucosa may become fragile and be accompanied with visible oozing (contact bleeding), bloody mucopurulent secretions may be attached, and multiple erosions, ulcers, and/or pseudopolyposis may be observed. However, the endoscopic diagnosis of UC is not always possible solely on the basis of these findings. These findings serve only as a standard to be referred to by gastroenterologists. One should also pay attention to the limitation that none of those endoscopic findings is specific to make a definite diagnosis of UC. Although histological findings of biopsy specimens in the active phase show diffuse inflammatory cell infiltration in the mucosal layer, crypt abscess, and remarkable goblet cell depletion, these findings are non-specific [14]. Abnormal crypt structures (distortion and branching) and atrophy persist in the remission phase. These findings are usually found continuously extending proximally from the rectum. Histological examination of biopsies is useful to diagnose cytomegalovirus infection complicating UC [24]. When the patient lacks endoscopic change in the rectum or the left side of the colon and a definite diagnosis is difficult to be established, histological examination is helpful to determine if a history of inflammation exists.

Endoscopic evaluation of the severity of intestinal disease is important to determine treatments of UC. Indices for the assessment of the endoscopic activity of UC include the Mayo endoscopic subscore and the Rachmilewitz index [25, 26]. The Mayo endoscopic subscore tends to be used in recent clinical trials; several reports from Western countries defined mucosal healing as score 0 (normal or inactive disease) or score 1 (mild disease: erythema, decreased vascular pattern, mild friability). Endoscopic evaluation of mucosal healing is useful to determine treatments of maintenance of remission and to predict relapse of UC, but there is an argument whether or not a score of 1 should be included in mucosal healing and it is necessary to analyze long-term data on this issue.

**CQ2-05. How should endoscopy be used for the diagnosis of CD?**

### Statements

- Colonoscopy (including observation of the terminal ileum) and histological evaluation of biopsies are recommended when a diagnosis of CD is suspected based on clinical findings (Recommendation grade: 1 (9), Evidence level: D).
- Upper gastrointestinal endoscopy had better be performed when a diagnosis of CD is suspected. Upper gastrointestinal endoscopy is recommended especially when lower gastrointestinal endoscopy fails to establish a definite diagnosis of CD, or patients complain of upper gastrointestinal symptoms (Recommendation grade: 1 (9), Evidence level: D).

### Comments

Inflammation of CD develops in any part of the gastrointestinal tract, but is more likely to develop in the colon and the lower ileum. Therefore, both barium contrast radiography and endoscopy are necessary to diagnose CD. Especially when clinical symptoms or laboratory examinations suggest CD, colonoscopy with the observation of the terminal ileum should be promptly conducted in order to establish a diagnosis, determine the extent and severity of the disease, and obtain biopsy specimens for a histological examination [21, 27–29]. Besides, when CT suggests inflammation in the small intestine in the pelvis, it is useful to perform not only conventional colonoscopy, but also a combination of endoscopic retrograde ileography in addition to an endoscopic observation of the terminal ileum under the X-ray. It is reported that when IBD is suspected, colonoscopic findings can distinguish CD from UC with an accuracy of 89% [29]. Characteristic colonoscopic findings of CD include discontinuous and regional lesions (so-called skip lesion), cobblestone appearance, longitudinal ulcers, irregular-shaped ulcers, multiple aphthous ulcers, narrowing and stenosis, (internal and external) fistula, and perianal lesions. The Crohn’s Disease Endoscopic Index of Severity (CDEIS) is proposed to be used as an index of endoscopic activity for CD; however, it is not suitable for use in daily practice because it is too complicated and time-consuming to calculate the scores [27]. Endoscopy is recently used not only to establish the diagnosis of CD, but also to treat stricture.

Upper gastrointestinal lesions are not rare in CD patients and are observed at a high rate (17–75%) regardless of the presence or absence of symptoms. Frequently observed upper gastrointestinal lesions of CD include a bamboo-like appearance in the stomach, gastric erosions/ulcers, duodenal erosions/ulcers, notch-like appearance and longitudinal ulcers in the duodenum. Since the Japanese diagnostic criteria for CD include irregular-shaped ulcer and/or aphtha observed both in the upper and lower gastrointestinal tract as a sub-criterion, upper gastrointestinal endoscopy is useful to assess lesions and obtain biopsy specimens for a histological examination (including the detection of non-caseous epithelioid cell granuloma) in order to establish a diagnosis of CD and exclude other diseases [18]. It is advisable to positively perform upper gastrointestinal endoscopy especially in patients in whom a definite diagnosis cannot be established with colonoscopy or who complain of upper gastrointestinal symptoms [21, 30]. Histological examination of CD focuses on the identification of granuloma, but the detection rate of granuloma in biopsy specimens is only 26–67% [31–33]. Moreover, it should be noted that granuloma may be observed in intestinal tuberculosis, infectious enterocolitis, and UC.

Capsule endoscopic investigation may be useful in patients in whom small intestinal lesions are suspected, but are unable to be detected with other examinations such as small bowel contrast imaging [34]. Capsule endoscopy (CE) is approved for clinical use in Japan in patients with the definite diagnosis of CD, although it is necessary to confirm the patency of the intestine in advance by a patency capsule. But, diagnostic criteria by CE or its usefulness based on evidence has not yet been established [35].

***2) Imaging examinations***

**CQ2-06. How should imaging examinations (except for endoscopy) be used for the diagnosis of UC?**

### Statements

- Ultrasonography (US), computed tomography (CT), and magnetic resonance imaging (MRI) are recommended to determine the activity before and after treatment or identify complications of UC (Recommendation grade: 1 (8), Evidence level: C).

### Comments

Endoscopy is a standard examination to diagnose UC and endoscopic and histological findings are most useful to establish the diagnosis (Table 1) [22, 36–39]. Barium enema examination is useful for determining the extent of UC and its activity in the deeper colon when endoscopy cannot reach there because of a stricture, etc. [37, 39]. Abdominal US, CT (including colonography), and MRI (including colonography) are also used for the same purpose, and they can provide information on not only luminal status, but also extraluminal lesions [37, 38]. According to a meta-analysis of prospective studies, it is reported that the sensitivity and specificity for the diagnosis of UC are 89.7%/95.6% by US, 84.3%/95.1% by CT and 93.0%/92.8% by MRI [40]. However, the adopted papers included many patients already diagnosed with IBD, therefore the usefulness of these examinations for establishing the definite diagnosis of UC is inconclusive [37, 41]. These imaging examinations are not stated in the Japanese diagnostic criteria for UC and are used as supportive examinations [36], therefore, the use of US, CT, and MRI for establishing the diagnosis of UC is limited. However, they are commonly used and useful for determining the activity of UC before and after treatment. It is necessary to choose optimal imaging modalities for the individual patient considering the characteristics of each examination since it is sometimes difficult to perform endoscopy in severe patients [22, 37].

**CQ2-07. How should imaging examinations (except for endoscopy) be used for the diagnosis of CD?**

### Statements

- Contrast radiography and other imaging examinations are recommended to determine the treatment strategy, disease extent, severity and complications of CD (Recommendation grade: 1 (9), Evidence level: C).
- US, CT, and MRI are recommended to be used mainly for evaluation of disease activity before and after treatment and complications of CD (Recommendation grade: 1 (8), Evidence level: C).

### Comments

It is extremely important to confirm the major (longitudinal ulcers and cobblestone appearance) and minor findings (irregular or oval ulcers, or aphtha observed in the extensive areas of the digestive tract) described in the Japanese diagnostic criteria for CD by contrast radiography (small bowel contrast imaging, barium enema examination, and endoscopic retrograde ileography) or endoscopy (Table 1) [18, 42]. In fact, it is reported that 87.4% of patients in Japan were diagnosed based on the major findings; i.e., longitudinal ulcers and cobblestone appearance [43]. There is no statement on imaging techniques such as US, MRI, and CT in the Japanese diagnostic criteria for CD [18, 42] and therefore these are used as supportive examinations. Nonetheless, the investigation of the gastrointestinal tract other than the colon including the upper gastrointestinal tract and small intestine is necessary even if the diagnosis of CD has been established based on the findings of colonoscopy or the barium enema examination since CD causes lesions throughout the entire gastrointestinal tract. To determine the therapeutic strategy, it is important to appropriately determine the disease extent (ileal/colonic/ileocolonic) and behavior (Montreal classification; non-stricturing non-penetrating/stricturing/penetrating). These should be confirmed by combining upper gastrointestinal endoscopy, a small bowel contrast study, a retrograde contrast study of the ileum, abdominal US, CT, and MRI, if necessary [22, 38, 44]. Although CE and balloon-assisted endoscopy (BAE) are being widely used for the evaluation of small intestinal lesions in CD, small bowel contrast imaging is still extremely important since CD frequently develops strictures [44]. There is no consensus on which examination should be used to establish the diagnosis of CD, although there are many reports comparing the usefulness of various imaging techniques [34, 40, 45]. A prospective comparative study reported that the sensitivity and specificity for the diagnosis of CD are 83%/53% by CE, 67%/100% by CT (enterography), 67%/100% by ileocolonoscopy, and 50%/100% by small bowel contrast imaging [46]. It is difficult to examine the small intestinal lesions by a single imaging technique; therefore, it is necessary to combine multiple examinations according to the pathophysiology of individual patients.

US, CT, and MRI can evaluate intestinal inflammation by the thickened bowel wall and increased fat density. CT and MRI are useful in examining fistula and abscess formation [45]. Enterography/colonography using CT or MRI are non-invasive and useful in evaluating the lesions beyond the strictures that cannot be evaluated by endoscopy, or perianal lesions [47, 48]. However, currently, these examinations are not feasible at every institution and there is no established protocol including pre-treatment (and medication) to inflate the bowel.

## 3. General considerations in treatment

**CQ3-01. Should patients with IBD quit smoking?**

### Statements

- Since smoking cessation is reported to have a negative effect on disease activity in UC patients, it is recommended that when recommending smoking cessation from the viewpoint of comprehensive health benefits, non-smoking policy should be promoted while paying attention to change in disease activity (Recommendation grade: 1 (8), Evidence level: C).
- It is recommended that non-smoking policy is promoted in patients with CD (Recommendation grade: 1 (9), Evidence level: C).

### Comments

According to the report by Beaugerie et al. [49] that included 32 patients who had stopped smoking after the diagnosis of UC and compared their prognosis between before and after smoking cessation, the periods of time with active disease, hospitalization, steroid use, and immunomodulator use were longer after stopping smoking than before stopping smoking. In addition, the administration period of these immunosuppressive therapies was significantly longer in ex-smokers than in continuing smokers [49]. In CD patients, smokers have a higher risk of surgery, postoperative clinical relapse, and reoperation compared to non-smokers [50, 51].

It is reported that in CD patients, smokers are more likely to reduce a response to infliximab (IFX) [52, 53].

In CD patients, regarding cigarette consumption, the periods of time with active disease and immunosuppressive therapy were reported to be longer even in light smokers who smoke less than 10 cigarettes per day than non-smoker patients [54].

In the study by Cosnes et al. [55] in which they intervened with CD patients by counseling and nicotine replacement therapy, it is reported that patients who successfully stopped smoking for over a year had less recurrence and less use of corticosteroids and immunomodulators than those who failed to stop smoking, and the risks were equivalent to patients who had never smoked.

**CQ3-02. Should patients with IBD quit drinking?**

### Statements

- It is recommended to appropriately advise patients with IBD to refrain from excessive alcohol consumption taking into consideration their medical condition such as disease activity and complications, even though there is no evidence to encourage abstinence from drink (Recommendation grade: 1 (8), Evidence level: D).

### Comments

There are few studies on an association between alcohol drinking and disease activity or prognosis in patients with UC and CD. However, it is reported that patients with IBD are more likely to complain about worsening of symptoms by alcohol drinking compared to patients with irritable bowel syndrome [56].

## 4. Therapeutic interventions for IBD

**CQ4-01. What are the risks/benefits and indications of 5-aminosalicyclic acid in the treatment of IBD?**

### Statements

- 5-Aminosalicylic acid (ASA) preparations are effective for induction and maintenance of remission in UC (Evidence level: A).
- The efficacy of 5-ASA preparations for CD is generally lower than UC. It is effective in reducing disease activity in active CD, whereas its efficacy for maintenance of remission has not been proven (Evidence level: B).
- The efficacy of 5-ASA preparations for preventing UC-associated CRC is inconclusive (Evidence level: B).

### Comments

There are many randomized clinical trials (RCT) and systematic reviews evaluating the efficacy and safety of oral and topical 5-ASA preparations for induction and maintenance of remission in UC [57–61]. Pentasa^®^ and Asacol^®^, the oral mesalazine preparations available in Japan, have a difference in the mechanisms of drug delivery to the lesion. Pentasa^®^ is a time-dependent slow-releasing medicine, whereas Asacol^®^ is pH-dependent slow-releasing medicine. There is a clinical trial directly comparing the efficacy in the treatment of UC between them, but the dose setting in the study was not appropriate to conclude which drug is superior to the other, and there is no clinically apparent difference.

Oral salazosulfapyridine (SASP) is as effective as oral mesalazine for induction of remission, but is superior in maintenance of remission [60, 61]. On the other hand, SASP causes side effects more frequently and patients are less tolerant to it than oral mesalazine when used to induce remission [60]. Attention must be paid to reversible male infertility by this drug. SASP is a compound in which 5-ASA and sulfapyridine are azo-bonded, and it exerts therapeutic effect after 5-ASA is released by cleavage of the azo bond by the action of intestinal bacteria. The other cleavage product, sulfapyridine, is considered to be responsible for many side effects of SASP, and therefore mesalazine, which is composed only of 5-ASA was developed.

There are much smaller numbers of clinical trials studying the efficacy of 5-ASA preparations in CD than UC. There are 2 RCTs in 1970s–1980s demonstrating the efficacy of SASP for induction of remission in CD; however, it is also shown that SASP is inferior to corticosteroids [62]. The meta-analysis including 3 studies on Pentasa^®^ reported that mesalazine is significantly more effective than placebo in reducing CDAI scores [63]; however, it is inconclusive whether or not the effect is clinically significant, and in addition, there was no significant difference in induction of remission compared to placebo [62]. The lack of efficacy of 5-ASA for maintenance of remission in CD is confirmed by the meta-analysis of placebo-controlled trials with a sufficient sample size [64]. A meta-analysis reported that 5-ASA preparations are effective to a certain degree for preventing postoperative relapse; however, it is not very conclusive since efficacy was not confirmed in 2 trials with a large sample size and there is a possibility of a publication bias [65].

There are several meta-analyses on the preventive effect of 5-ASA preparations for carcinogenesis. The results were split to positive [66, 67] or negative [68] conclusions and inconclusive.

**CQ4-2. What are the risks/benefits and indications of corticosteroids in the treatment of IBD?**

### Statements

- Corticosteroids have potent anti-inflammatory property and are effective for induction of remission in UC and CD (Evidence level: B).
- Corticosteroids have no efficacy for maintenance of remission and their long-term use can lead to adverse events; therefore, they should not be used for maintenance of remission (Evidence level: C).

### Comments

Several RCTs have demonstrated an efficacy of corticosteroids alone for inducing remission in both UC and CD since 1960s in Europe and the US, and a few meta-analyses also showed that corticosteroids are more effective for inducing remission compared to placebo [69, 70]. However, one should keep in mind that the quality of the meta-analyses was not high because most RCTs that the meta-analyses reviewed are old and there is heterogeneity in disease severity, disease extent, the types of steroids, dosing regimens, allocation, etc. Appropriate indications of corticosteroids, including disease severity and type, needs to be determined by further studies since new treatments such as novel forms of 5-ASA preparations and anti-TNF agents have been developed.

It has been emphasized repeatedly that corticosteroids are not effective for maintenance of remission. This is proven in CD by a meta-analysis [71], but there are only two old RCTs that proved the lack of efficacy of corticosteroids for maintenance of remission in UC [72, 73]. Long-term or high-dose use of steroids should be avoided and they should not be used to maintain remission because of their various side effects such as immunosuppression, impaired glucose tolerance, a delay in wound healing, and osteoporosis. It is necessary to withdraw and discontinue corticosteroids after determining their efficacy even when they are used for inducing remission, but there is no clear evidence on how to withdraw them. When high-dose or unavoidable long-term administration of corticosteroids is inevitable, attention should be paid to cataract, glaucoma, and adrenal cortical insufficiency, and prophylaxis of pneumocystis pneumonia and prevention of osteoporosis by bisphosphonate are advisable (details in the Guidelines on the Management and Treatment of Glucocorticoid-induced Osteoporosis of the Japanese Society for Bone and Mineral Research: 2014 update) [74].

Budesonide, which has reduced systemic side effects compared to conventional corticosteroids (e.g., prednisolone), is effective for inducing remission in CD, but its efficacy is slightly lower than conventional corticosteroids [75]. Moreover, its efficacy for maintenance of remission is negative [76].

Besides oral administration, intravenous systemic administration of corticosteroids is used, but there is no clear evidence on it. Furthermore, rectal administration of corticosteroids (enema, suppository) is also effective as a treatment of UC; however, rectal administration should not be chosen as first-line therapy because its efficacy for induction of remission is lower than 5-ASA preparations.

**CQ4-03. What are the risks/benefits and indications of immunomodulators in the treatment of IBD?**

### Statements

- Azathioprine (AZA)/6-mercaptopurine (6-MP) are effective for preventing relapse in UC patients in remission, and therefore are effective for maintenance of remission especially in patients who are steroid-dependent or unable to maintain remission by 5-ASA preparations (Evidence level: A).
- AZA/6-MP are effective for maintenance of remission in CD. Administration of AZA/6-MP are effective to avoid surgery, and they are also effective to prevent from postoperative clinical and endoscopic relapse. Their combined use with IFX increases the efficacy for inducing remission compared to IFX alone (Evidence level: A).
- Use of AZA/6-MP increases the risk of developing lymphoma. In addition, other side effects include gastrointestinal symptoms such as nausea, myelosuppression, alopecia, and pancreatitis (Evidence level: A).
- Tacrolimus (TAC) is effective for inducing remission in active UC; however, there is no sufficient data on the efficacy and safety of its long-term use (Evidence level: C).
- Cyclosporine (CyA) is effective as a remission induction treatment in severely active and refractory UC and is as effective as IFX (Evidence level: C).

### Comments

The first RCT examining the efficacy of AZA/6-MP for maintenance of remission in UC was published in 1970s; however, there are only 4 placebo-controlled RCTs [77, 78]. Their efficacy was rather assessed mostly by the long-term experience on their use and retrospective studies about them.

There are more data demonstrating the efficacy of AZA/6-MP for maintaining remission in CD compared to UC [79, 80]. Several prospective studies examining their effect on postoperative recurrence have been conducted since the 2000s and demonstrated their efficacy in preventing clinical and endoscopic relapse [81]. A review of more than 10 retrospective studies concluded that it is useful in avoiding the first surgery [80]. A prospective placebo-controlled study (SONIC study) demonstrated that a combination treatment of AZA with IFX is superior to IFX alone in terms of the rate of induction of remission in CD [82].

The increased risk of developing lymphoma by AZA/6-MP has been confirmed by a meta-analysis [83], a multicenter large-scale cohort [84], and the US nationwide cohort [85]. According to these studies, the risk is reported to increase approximately 4 times, but decreases again after discontinuation of AZA/6-MP. A combined use of AZA/6-MP with IFX is reported to induce the development of fatal hepatosplenic lymphoma, although it is rare. There are 40 reported cases of this type of lymphoma among IBD patients worldwide. Most of the patients are males under the age of 35 years [86]. Another important side effect of AZA/6-MP includes myelosuppression and the rate of developing severe myelosuppression in which neutrophil count is less than 1000/μl is reported to be ~ 1% [87].

There are only 2 RCTs confirming the efficacy of TAC for inducing remission in UC, which are the phase 2 and 3 clinical trials conducted in Japan [88, 89]. There is only 1 small-size placebo-controlled RCT examining the efficacy of CyA for induction of remission in severe steroid-refractory UC [90]. A recent head-to-head trial indicated that CyA is as effective as IFX in steroid-refractory UC [91]. Both Tac and CyA may induce nephrotoxicity as a side effect.

**CQ4-04. What are the risks/benefits and indications of antibiotics and probiotics in the treatment of IBD?**

### Statements

- Antibiotics may be effective for induction of remission in CD (Evidence level: C).
- Antibiotics can reduce discharge from anal fistula in CD (Evidence level: B).
- Antibiotics may be effective for induction of remission in UC, but the type and duration of antibiotics to use are not established (Evidence level: C).
- Antibiotics are also effective for pouchitis after colectomy for UC (Evidence level: B).

### Comments

Administration of antibiotics, probiotics, and prebiotics have been examined as treatments for IBD because the intestinal microbiota is suggested to be associated with the development of IBD [92]. Those treatments are conducted to control the intestinal microbiota as an aggravating factor in IBD and to treat or prevent from bacteremia, abscess, and opportunistic infection. Apart from these effects, it is suggested that particular antibiotics (ex. ciprofloxacin (CPFX), metronidazole (MNZ), macrolide antibiotics) possibly affect IBD as immunomodulators [93].

Two meta-analyses reported that monotherapy with CPFX or MNZ, or the combination of the two antibiotics are effective for induction of remission in active CD [94, 95]; however, specific indications and administration protocols of these antibiotics have not yet been determined. A meta-analysis showed that administration of CPFX or MNZ decreased discharge from anal fistula in CD patients [94].

Two meta-analyses reported a low efficacy of antibiotics for UC [95, 96], but types and administration period of antibiotics were different among RCTs, and, therefore, antibiotics are not recommended as remission induction therapy for UC. In the previous therapeutic regimen of high-dose intravenous steroid, short-term empiric administration of broad-spectrum antibiotics was recommended [97]; however, the efficacy of antibiotics for severe UC has not been proved, and, therefore, its long-term use should be avoided even when administered on the suspicion of a complication of infection. It is reported from Japan that the combination therapy of three antibiotics (ATM) is effective for UC [98].

Use of CPFX or MNZ alone or their combination is effective for pouchitis after colectomy for UC and is a standard treatment for it (refer to CQ4–8) [99].

Attention should be paid to peripheral neuropathy as a side effect of MNZ especially when it is administered for a long period and at a high dose. CPFX causes fewer side effects and are more tolerable.

Regarding the administration of probiotics for IBD, three meta-analyses on VSL#3 or *E coli* Nissle 1917 (unreleased in Japan) demonstrated that VSL#3 is effective for induction and maintenance of remission in UC but probiotics did not show efficacy for CD or pouchitis [100–102].

**CQ4-05. How effective are anti-TNF agents in the treatment of IBD?**

### Statements

- Anti-TNF agents are effective for induction of remission in steroid-refractory or steroid-dependent moderate-to-severe UC (Evidence level: A).
- Anti-TNF agents are effective for induction and maintenance of remission in patients with CD with active inflammation (Evidence level: A).

### Comments

#### Efficacy in UC

According to meta-analyses and RCTs, IFX [103] and adalimumab (ADA) [104] are effective for induction of remission in steroid-refractory or -dependent moderate-to-severe UC. It is reported that secondary loss of response may develop in about 60% of the patients who initially respond to anti-TNF agents during approximately 5-year follow-up [105].

#### Efficacy in CD

A meta-analysis [103] in 2011 showed that both IFX and ADA were effective for induction of remission in CD patients with active inflammation. Although IFX is effective for preventing relapse of CD patients in remission, the efficacy of ADA for preventing relapse is not confirmed. A multicenter RCT involving a total of 52 institutions in the US, Canada, Belgium, and France demonstrated that ADA, which was administered at the initial dose of 160 mg, followed by the dose of 80 mg after 2 weeks, was effective for inducing remission in moderate-to-severe CD patients who were intolerant to IFX or lost response to IFX [106]. The RCT enrolling moderate-to-severe CD patients who were steroid-dependent or refractory to high-dose mesalazine or steroids showed that combination therapy with IFX and AZA was significantly superior to IFX alone in the clinical remission rate at 26 weeks [107]. It is unknown how long anti-TNF therapy should be continued in CD patients who achieved remission with the combination therapy of anti-TNF agents and AZA. However, the prospective cohort study with 115 CD patients in remission in 20 institutions from Belgium and France showed that 50% of the patients relapsed within a year after discontinuation of IFX [108]. Anti-TNF agents were reported to be effective for anal fistula in CD [109].

#### Side effects of anti-TNF agents

Reactivation of infections such as tuberculosis and hepatitis B by administering anti-TNF agents has been reported [110, 111]. Therefore, it is critical to confirm the absence of latent tuberculosis and hepatitis B virus infection prior to starting anti-TNF therapy. A tuberculin skin test and an interferon-gamma release assay in addition to chest X-ray should be done to exclude latent lung tuberculosis [1]. Regarding hepatitis B virus infection, tests for HBs-Ag, anti-HBs-Ab, and anti-HBc-Ab should be done [112]. According to a retrospective observational study, skin lesions developed in approximately 30% of patients who were administrated anti-TNF agents [113]. Demyelinating disease and peripheral neuropathy were also reported.

Regarding side effects of IFX used for induction of remission of UC, a meta-analysis in 2011 showed no statistically significant differences in the incidence of either abnormal responses to infusion (infusion reaction or injection site reaction), headache, skin lesions, or arthralgia compared with the placebo group [103]. Furthermore, as for side effects of anti-TNF agents used for induction of remission in active CD patients, there was no statistically significant difference in the incidence of either infection, injection site reaction, headache, abdominal pain, nausea/vomiting, arthralgia/myalgia, or fever compared with the placebo group.

The prospective cohort study including 6000 CD patients in North America reported that IFX was not associated with severe infection (adjusted OR 0.991, 95% CI 0.641–1.535) [114]. Additionally, the prospective observational study using the same cohort suggested that the adjusted hazard ratio for the development of malignancy by IFX monotherapy was 0.59 (95% CI 0.28–1.22) [115], and IFX monotherapy was not a significant risk factor for malignancy. However, combination therapy with IFX and thiopurine is a risk factor for non-Hodgkin lymphoma [116] and hepatosplenic lymphoma [117]. Moreover, the RCT aiming to determine the efficacy of IFX showed that antinuclear antibody and anti-DNA antibody developed more frequently in the IFX group compared with the placebo group [118]. An RCT investigating the efficacy of ADA reported that the incidence of injection site inflammation and leukocytopenia was higher in the ADA group than the placebo group [104].

**CQ4-06. What are the risks/benefits and indications of nutrition therapy in the treatment of IBD?**

### Statements

- Efficacy of nutrition therapy alone including enteral nutrition and total parenteral nutrition for inducing remission in UC has not been confirmed; therefore UC patients should not easily be forced to restrict diet and treatment should focus on drug therapy and/or cytapheresis (CAP) (Evidence level: C).
- Enteral nutrition therapy is effective for inducing remission in patients with active CD. Enteral nutrition therapy has a good safety profile, but is occasionally difficult for patients to accept (Evidence level: C).
- Elemental diet is effective for maintaining remission in CD (Evidence level: B).

### Comments

Nutrition therapy (enteral nutrition therapy, total parenteral nutrition, etc.) is not effective for induction of remission in UC patients [119], unlike CD patients. Although nutritional management is necessary for the acute stage of UC, it is not appropriate to use nutrition therapy for induction of remission [22]. Unlike CD, there is no evidence showing the efficacy of diet therapy or home nutrition therapy for UC patients in remission. Many UC patients tend to voluntarily have dietary restrictions and avoid dairy products even when they are in remission [120]. However, it is unknown that these restrictions are effective for preventing relapse, on the contrary, it may cause a nutritional deficiency of calcium and so on [120]. UC patients, especially those in remission, should not easily restrict their diet because the restriction may possibly disturb nutritional status and decrease QOL.

A meta-analysis showed that the efficacy of enteral nutrition therapy for induction of remission in active CD patients is inferior to corticosteroids (OR 0.3, 95% CI 0.17–0.52) [121]. For this reason, in Western countries, nutrition therapy is only used as an alternative to corticosteroids or in order to improve nutritional status in the acute stage in adult CD patients [2, 22]. On the other hand, a Japanese study reported that enteral nutrition therapy with elemental diet has a higher rate of induction of remission in CD patients compared with corticosteroids, and especially improves luminal lesions [122], therefore it is chosen as an option of remission induction therapy in CD patients in Japan [123]. Enteral nutrition therapy is superior to drug therapy such as corticosteroids in terms of safety. However, it may be difficult for some patients to continue it due to low tolerability [124]. Since oligomeric formula is an enteral nutrient formula with amino acids or oligopeptides as nitrogen sources and a little fat content, it is easy to absorb and digest in the bowel. Among oligomeric formulas, an elemental formula utilizes amino acids as a source of nitrogen and contains a very little amount of fat. Polymeric formula is an enteral nutrient formula which contains protein as a nitrogen source and some fat. Polymeric formula is well-balanced in nutrients and easier to ingest orally. There have been many RCTs comparing the efficacy of various nutritional therapies for induction of remission in active CD, but a meta-analysis concluded that there is no significant difference between polymeric and oligomeric formulas in the efficacy for induction of remission [121]. Therefore, in clinical settings in Japan, these treatment options are selected on an individual basis considering acceptability and preference of each patient, although there seems to be no significant difference between these formulas in efficacy for induction of remission.

Enteral nutrition therapy is effective not only for induction of remission, but also for maintenance of remission in CD. It is reported that ingestion of half calories of the total calorie intake with elemental diet is more effective for maintenance of remission compared to dietary counseling alone [125]. However, it has been pointed out that there is a problem in acceptability as therapy despite its proven efficacy [126]. These 2 trials studying maintenance of remission utilized different evaluation methods although each study demonstrated the efficacy of nutrition therapy [127]. Long-term remission maintenance therapy is necessary for CD patients because there is no cure for CD, but it is not easy to continue enteral nutrition therapy. In clinical practice, nutrient formulas other than oligomeric nutrient formula such as polymeric nutrient formulas are used, considering the acceptability; however, their effects on maintenance of remission have not been well evaluated yet [128].

It is recently expected that enteral nutrition therapy may be more effective when combined with drug therapy, especially anti-TNF agents; however, there are no high-quality clinical studies to prove it.

**CQ 4-07. What are the risks/benefits and indications of CAP in the treatment of IBD?**

### Statements

- CAP is a useful remission induction therapy for moderate-to-severe UC patients and has a good safety profile. Intensive therapy (two sessions per week) provides more rapid induction of remission with a better remission rate than weekly therapy (Evidence level: C).
- In CD patients with active colonic disease, if pharmacotherapy or nutrition therapy is ineffective or unable to adapt, the combination with granulocyte monocyte apheresis (GMA) can be considered (Evidence level: D).

### Comments

There are two methods of CAP, GMA (Adacolumn^®^) and leucocytapheresis (LCAP; Cellsorba^®^), for UC in Japan (as of 2016). GMA is filled with specially designed cellulose acetate beads as the adsorptive carriers, which selectively adsorb granulocytes and monocytes, while LCAP uses the leukocyte removal filter made of polyester non-woven fabric, which removes leukocytes including lymphocytes and platelets. There is no clear evidence of a difference in efficacy and distinctive use of these two methods.

CAP is a treatment covered by insurance in Japan for moderate-to-severe UC patients. A blinded RCT using sham columns conducted in the US and Europe reported that GMA did not demonstrate significant therapeutic efficacy for induction of remission [129]. On the other hand, there is a meta-analysis examining the efficacy of CAP for induction of remission in moderate-to-severe UC patients, and it reported that CAP was superior to conventional pharmacotherapy in terms of safety and its steroid-sparing effect [130]. It is also reported that CAP produces high efficacy in steroid-naïve patients [131] and, therefore, it may be applied to patients who are naive to steroids, although there are no controlled trials.

An RCT (open label) in Japan reported that intensive GMA (two sessions per week) provides more rapid remission induction and a higher remission rate than weekly GMA (54 vs 71%) [132].

The usefulness of GMA for active colonic CD has been reported in patients who are refractory to conventional medical and/or nutritional therapies [133]. It was approved in 2010 for use in the treatment of CD in Japan.

CAP is a safe treatment with few side effects; however, sufficient blood flow cannot be secured in patients who are difficult to assure peripheral vascular access (including dehydrated and anemic patients), and CAP may be difficult to conduct in such patients. It is also known in clinical practice that severe patients who demonstrate extensive ulcers tend to show a poor response to CAP.

Efficacy of CAP for maintenance of remission in UC has been reported [134], although its evidence level is low and its use has not yet been approved for this purpose. There is only one case report suggesting its efficacy for maintaining remission in CD [135].

**CQ4-08. What are the risks/benefits of surgery in the treatment of IBD?**

### Statements

- Surgery can improve the life prognosis of patients with severe disease or co-existing dysplasia/cancer. In addition, it may also provide a better QOL for patients who are suffering from medically refractory IBD symptoms, side effects of drugs, or extraintestinal manifestations (Evidence level: D).
- Surgical procedures may cause postoperative complications such as anastomotic leak, intestinal obstruction, pouchitis in UC patients, and small intestinal failure in CD patients (Evidence level: D).

### Comments

In both UC and CD, patients who develop a severe disease refractory to drug therapy or have dysplasia/cancer require surgery to avoid life-threatening conditions (absolute indications) [22, 136, 137].

Patients who are suffering from the loss of QOL due to IBD symptoms, extraintestinal manifestations, or side effects of drugs should be also indicated for surgery [137], and improvement of QOL may be expected through the resolution of these symptoms after surgery [138–142].

However, there are risks and disadvantages of surgery, although the postoperative mortality is low in both diseases [143–145].

The standard surgical procedure for UC is total restorative proctocolectomy with ileal pouch-anal (canal) anastomosis. It may cause postoperative complications such as anastomotic leak, intestinal obstruction, and pouch-related complications [144]; however, the incidence of pouch failure (requiring excision of the ileoanal pouch, formation of a permanent ileostomy) is approximately 5% [146]. There are some reports suggesting a decrease in fertility in females, while the course and outcome of pregnancy are generally normal and defecation function is not worsened by pregnancy [147].

The incidence of postoperative anastomotic leak in CD patients is 2–14% [148, 149]. Furthermore, re-surgery due to recurrence of intestinal lesions may lead to small intestinal failure as a result of the shortening of the residual bowel, and total parenteral nutrition may be necessary for such patients [150].

## 5. Treatments of UC

**Fig. 3:**
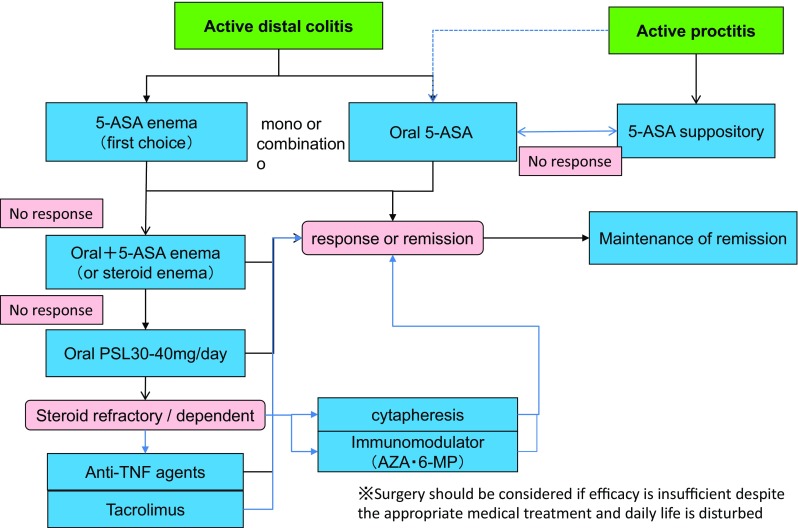
Remission induction treatment for mildly-to-moderately active distal UC

***1) Treatment for mildly to moderately active distal UC (Fig.*** ***3******)***

**CQ5-01. What are the indications of 5-ASA in the treatment of mildly to moderately active distal UC?**

### Statements

- It is suggested to use 5-ASA enema as first-line therapy for induction of remission (Recommendation grade: 2 (7), Evidence level: B).
- Oral 5-ASA alone is also effective and is recommended for induction of remission (Recommendation grade: 1 (9), Evidence level: A).
- Combination of oral and topical 5-ASA is recommended for patients requiring more potent treatment than oral or topical 5-ASA alone (Recommendation grade: 1 (9), Evidence level: B).
- 5-ASA enema is recommended as a first choice of topical treatment because it is at least comparable to or superior to steroid enema therapy in terms of efficacy (Recommendation grade: 1 (8), Evidence level: B).

### Comments

Many RCTs and meta-analyses demonstrated that both topical and oral 5-ASA are effective for induction of remission [58, 60, 151]. There are only a few RCTs and meta-analyses comparing the efficacy between oral and topical 5-ASA; some reported that topical therapy is more effective than oral therapy and others reported that there is no significant difference between oral and topical therapies [58, 152–154]. It is necessary to choose the treatment considering factors including mucosal concentrations of 5-ASA and drug adherence of patients; however, many guidelines in the US and Europe recommend topical 5-ASA as first-line therapy [1, 136]. Oral medications are more likely to be favored in Japan compared to topical medications because topical medications are more time- and effort-consuming to administer than oral medications, which may lead to decreased drug adherence. The Research Group for Intractable Inflammatory Bowel Disease positions oral 5-ASA in parallel with topical 5-ASA.

As oral 5-ASA preparations, conventional SASP and two types of mesalazine with different release mechanisms in the intestinal tract are available in Japan; however, according to a meta-analysis, there is no difference in the efficacy between SASP and mesalazine. However, SASP may not be tolerated or may cause side effects, therefore mesalazine tends to be favorably used in Japan; on the other hand, SASP is more recommended especially in the US because of its lower cost. There is no difference in efficacy between the two types of mesalazine preparations.

Since there is no significant difference in the efficacy of 5-ASA enema among the daily dose levels of 1, 2, and 4 g, the dose level of 1 g/day is sufficient [152]. A dose–response relationship is observed in oral 5-ASA; dose levels of 2 g/day or more provide a significantly higher rate of induction of remission than dose levels of less than 2 g/day. Although there is no clear evidence on the efficacy of high doses of 4.0 g/day or more because endpoints varied among studies, a higher dose is desirable for moderately active UC patients [60].

It has been confirmed that the combination of oral and topical 5-ASA therapy significantly improves efficacy compared to using either agent alone [57]. Therefore, the combination therapy is recommended for patients showing poor response to monotherapy of either oral or topical 5-ASA or those with severe symptoms. Some guidelines recommend the combination therapy from the beginning, with an expectation of rapid alleviation of the symptoms.

It is demonstrated that 5-ASA enema is superior to steroid enema [58], therefore 5-ASA enema should be used as first-line therapy. When patients show a poor response to 5-ASA enema or combination with oral 5-ASA, steroid enema therapy should be considered.

**CQ5-02. What are the indications of corticosteroids in the treatment of mildly to moderately active distal UC?**

### Statements

- It is recommended that neither oral or topical steroids should be selected as first-line therapy even though these drugs are effective for induction of remission (Recommendation grade: 1 (9), Evidence level: A).
- It is recommended not to use steroid enema as first-line therapy because it is comparable or inferior to 5-ASA enema in terms of efficacy (Recommendation grade: 1 (8), Evidence level: A).
- In patients who do not respond to oral 5-ASA therapy at an optimal dose combined with topical 5-ASA or steroid therapy, it is recommended that oral prednisolone (PSL) is started at a daily dose of 30–40 mg (Recommendation grade: 1 (9), Evidence level: B).

### Comments

Both steroid enema therapy and oral steroid are effective for inducing remission in patients with active distal colitis [69, 155]. Budesonide enema and betamethasone dipropionate (BDP) enema, which have a low risk of systemic side effects, are available overseas but not in Japan.

There is a meta-analysis showing that steroid enema therapy is comparable to 5-ASA enema therapy in terms of efficacy for mild-to-moderate patients with active distal colitis [156]; on the other hand, there is another meta-analysis showing that 5-ASA enema is superior to steroid enema [58, 155]. The steroid used in the former meta-analysis is BDP, which is not available in Japan.

It has been confirmed that the combination of oral 5-ASA and enema therapy is more effective for active distal colitis compared to either agent alone; however, when patients do not respond to oral 5-ASA therapy at an optimal dose combined with topical therapy, oral steroids are indicated. Steroids had been introduced in the treatment of UC long before the quality of clinical trial design was strictly required. Evidence on the efficacy of steroid therapy for active distal colitis is scarce. A meta-analysis examining the efficacy of oral steroids in inducing remission in active UC including extensive colitis demonstrated that oral steroid therapy is superior to placebo [69]. UC patients have various physical and nutritional conditions; therefore, it is advisable that the dose of steroids should be adjusted in each patient referring to the dose shown here.

Either steroid enema or oral steroids are not effective for maintenance of remission.

**CQ5-03. What are the indications of miscellaneous treatments in the treatment of mildly to moderately active distal UC?**

### Statements

- It is recommended that use of CAP or IFX/ADA should be considered for UC patients who do not respond to 5-ASA preparations or steroids (Recommendation grade: 1 (9), Evidence level: C).
- Antibiotics may be effective for induction of remission but appropriate types of antibiotics, their combinations, and treatment duration have not been determined (Evidence level: C).

### Comments

When patients with mildly to moderately active distal colitis do not respond to conventional 5-ASA or steroid therapies, remission induction therapies used for moderate-to-severe patients should be considered. Patients are usually outpatients, therefore, anti-TNF agents such as IFX and ADA, or CAP will be the next therapy [1] (refer to CQ5–10 and CQ5–11 for detail). Evidence has proven the efficacy of these therapies for moderate-to-severe colitis; however, there are few studies where subjects are limited to patients with mildly to moderately active distal colitis.

A meta-analysis indicated that antibiotics are effective for induction of remission unless the subjects are limited to distal colitis; however, the type and treatment duration of antibiotics varies among RCTs; therefore, antibiotics have not yet been recommended as remission induction therapy [94]. The results of an RCT studying the efficacy of a combination of 3 antibiotics have been reported from Japan, but confirmatory studies have not been conducted.

It is impossible to evaluate the efficacy of probiotics that are available in Japan because of the absence of sufficient data. Additionally, a meta-analysis failed to demonstrate the efficacy of probiotics for induction of remission [157]. In contrast, there are some RCTs which showed the efficacy of VSL#3 and *E coli* Nissle 1917 (unreleased in Japan) for induction of remission.

Furthermore, the remission induction effects of miscellaneous treatments such as fish oil, heparin, or nicotine were indicated by RCTs, although meta-analysis did not show the efficacy of these treatments superior to conventional therapies and neither of them are available in Japan [158–160].

**CQ5-04. What are the treatments of mildly to moderately active proctitis?**

### Statements

- 5-ASA suppository is recommended for induction of remission in patients with proctitis (Recommendation grade: 1 (8), Evidence level: B).
- When patients do not respond to 5-ASA suppository, it is recommended to consider combination therapy with oral 5-ASA or switch to topical steroid therapy (Recommendation grade: 1 (9), Evidence level: B).

### Comments

The effectiveness of topical 5-ASA therapy for distal colitis has been established [58, 152–154]; however, there is only a little evidence that was demonstrated by meta-analyses when limited to patients with proctitis or the efficacy of 5-ASA suppository. However, an RCT from Japan recently reported that mesalazine suppository was more effective than placebo [161]. There are only a few comparative studies between enema and suppository, but it is reported in 1980s that the efficacy of these therapies is comparable [162]. Many guidelines recommend the use of mesalazine suppository for patients with proctitis as first-line therapy considering sufficient drug delivery to the affected area and drug adherence of patients [136].

In addition to mesalazine suppository, SASP suppository is also available in Japan, but there has been little high-quality evidence for it and it has not been confirmed that SASP suppository is superior to mesalazine suppository in terms of efficacy; therefore, this guideline does not positively recommend the use of SASP suppository.

Evidence on enema preparation indicates that the efficacy of topical mesalazine does not increase in a dose-dependent manner above 1 g/day [152]. The efficacy of 1 g mesalazine suppository once daily is comparable to that of two doses of 500 mg [163]; therefore, the dose of 1 g once daily is recommended in terms of drug adherence.

There is a report that oral 5-ASA alone is less effective than mesalazine suppository [164], and when an oral preparation is used, it is desirable to combine it with a topical preparation or to use a high dose of pH-dependent-release mesalazine [136, 165].

Treatment options in patients who show an insufficient response to mesalazine suppository is extrapolated from evidence for distal-to-left-sided colitis [136]. 5-ASA enema may be an option although there is no evidence for its use in patients who are refractory to mesalazine suppository. Although the efficacy of topical steroid preparations is inferior to that of topical 5-ASA preparations [58], corticosteroid suppository (e.g., betamethasone suppository) may be effective in patients who show an insufficient response to mesalazine suppository.

Oral corticosteroids, anti-TNF agents, and/or immunomodulators should be considered as therapeutic options for proctitis resistant to the above treatments while considering the possibility of other pathophysiology such as infections that need to be differentiated from UC [136].

**Fig. 4:**
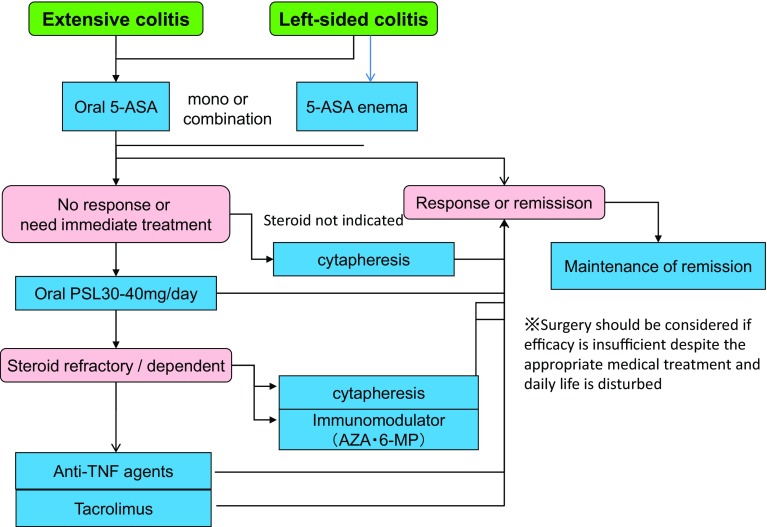
Remission induction treatment for mildly-to-moderately active extensive (including left-sided) UC

***2) Treatment for mildly to moderately active extensive UC (Fig.*** ***4******)***

**CQ5-05. What are the indications of oral 5-ASA in the treatment of mildly to moderately active extensive UC?**

### Statements

- It is recommended to use oral 5-ASA as first-line therapy (Recommendation grade: 1 (9), Evidence level: A).
- It is recommended to use 5-ASA enema for left-sided colitis (Recommendation grade: 1 (8), Evidence level: A).

### Comments

First-line therapy for extensive UC with mild-to-moderate activity is oral 5-ASA preparations including SASP. The efficacy of these drugs for inducing remission is confirmed by the Cochrane review [60]. The efficacy of 5-ASA preparations is dose-dependent and high-dose (3 g/day or more) is superior to low-dose (2–2.9 g/day) for induction of remission. There is no difference in the efficacy for induction of remission or endoscopic improvement between oral 5-ASA and SASP. However, a safety profile of SASP is inferior to 5-ASA due to the higher incidence of adverse events, although SASP is more cost-effective. Most side effects of SASP are thought to be due to sulfapyridine bound to 5-ASA. Its common side effects are skin rash, headache, epigastric discomfort, and male infertility.

There are two different forms of 5-ASA preparations available in Japan, time-dependent (Pentasa^®^) or pH-dependent-release (Asacol^®^) mesalazine; however, efficacy and safety do not differ between them if given in the same dose. Furthermore, its efficacy, safety, and acceptability are comparable between different dosing frequencies with once-daily and 2–3 times a day. Regarding the correlation between doses of 5-ASA and therapeutic effect, there are many reports suggesting the efficacy for induction of remission and safety is not dose-dependent if evaluated using the same preparation. However, the RCT of time-dependent mesalazine, which is available in Japan, for moderately active UC demonstrated that 4 g/day dosing showed a higher rate of clinical response than 2.25 g/day [166]. In addition, the large-scale RCT of pH-dependent-release mesalazine also suggests that high-dose is more effective than low-dose in patients with moderately active UC with a past history of treatment such as steroids [167]. It is recommended to start with a high-dose 5-ASA for moderately active UC since most data suggest no correlation between doses of 5-ASA and incidence of side effects.

5-ASA enema is also effective in active left-sided UC and has efficacy for clinical improvement and induction of remission. A meta-analysis of multiple RCTs confirmed the superiority of 5-ASA enema to oral preparations [152]. 5-ASA enema is reported to be superior to steroid enema in both efficacy and safety.

**CQ5-06. What are the indications of corticosteroids in the treatment of mildly to moderately active extensive UC?**

### Statements

- It is recommended to use 30–40 mg/day of PSL when the patient does not respond to the sufficient dose of 5-ASA (Recommendation grade: 1 (9), Evidence level: C).
- It is recommended to consider withdrawal of the steroid when a clinical response is observed and avoid its long-term use (Recommendation grade: 1 (9), Evidence level: D).

### Comments

Efficacy of steroids for UC has been demonstrated by a meta-analysis [69], but the quality of evidence is not very high since most studies are old and the doses/types of steroids vary widely among the studies. The optimal dose of steroids for mild-to-moderate UC has not been confirmed yet and therefore 40 mg/day of PSL is widely accepted overseas as a standard dose [22]. In contrast, 30 mg/day of PSL is frequently used in Japan, but it is unclear whether a dose of 30 mg/day is better than 40 mg/day in terms of risks/benefits. It is demonstrated that 20 mg/day dosing is less effective than 40 mg/day dosing [168].

There is no report on the optimal dosing period and the optimal tapering method of steroids after achieving remission, and guidelines in other countries just state the empirical rules [1, 169]. It is generally recommended to evaluate the response within 1–2 weeks and, after achieving clinical remission, reduce the dose of steroids at 5 mg/week until 20 mg/day and then at 2.5 mg/week.

**CQ5-07. What are the indications of miscellaneous treatments for the treatment of mildly to moderately active extensive UC?**

- It is recommended to use CAP for patients who are steroid-refractory, -dependent, or intolerant to steroids (Recommendation grade: 2 (7), Evidence level: B).
- It is recommended to consider TAC or anti-TNF agents for patients who are steroid-refractory or -dependent with moderate-to-severe activity (Recommendation grade: 1 (8), Evidence level: C).

### Comments

The meta-analysis regarding the efficacy of CAP for induction of remission in UC, which includes many publications from Japan, reported its good efficacy and safety [130]. In addition, an RCT conducted in Japan demonstrated that twice-weekly intensive treatment induces remission more rapidly compared with weekly treatment [132]. It may be effective in steroid-refractory or -dependent patients; moreover, it is reported that the response rate in steroid-naïve patients is even higher despite the lack of comparative studies [131]. Therefore, use of CAP can be considered for patients who are unable to use steroids for some reason.

There are only 2 placebo-controlled RCTs to assess the efficacy of TAC in UC, which were conducted in Japan and included steroid-refractory or -dependent patients [88, 89]. These trials demonstrated high-trough dosing (10–15 ng/ml) is more effective than low-trough dosing (5–10 ng/ml). The currently recommended protocol is to administer the drug twice daily orally, adjust the trough levels to 10–15 ng/ml during the first 2 weeks, and then reduce the trough levels to 5–10 ng/ml.

The efficacy of anti-TNF agents (IFX and ADA) for induction and maintenance of remission until week 52 in refractory UC has been confirmed in placebo-controlled trials [170, 171], while there is no head-to-head trial directly comparing IFX and ADA. IFX is intravenously administered every 8 weeks following induction dosing at 0, 2, and 6 weeks. ADA is administered subcutaneously at the initial dose of 160 mg, followed by 80 mg at week 2 and then 40 mg every other week.

There are a few placebo-controlled trials to examine the efficacy of antibiotics and a meta-analysis also indicated efficacy of antibiotics; however, its reliability as a meta-analysis is to be debated because the types and doses of antibiotics vary across the studies [94]. Furthermore, it is impossible to discuss the efficacy of the probiotics available in Japan because of the absence of sufficient data, while there are some RCTs confirming the efficacy of VSL#3 and *E. coli* Nissle 1917 that are commercially available overseas [172].

**Fig. 5:**
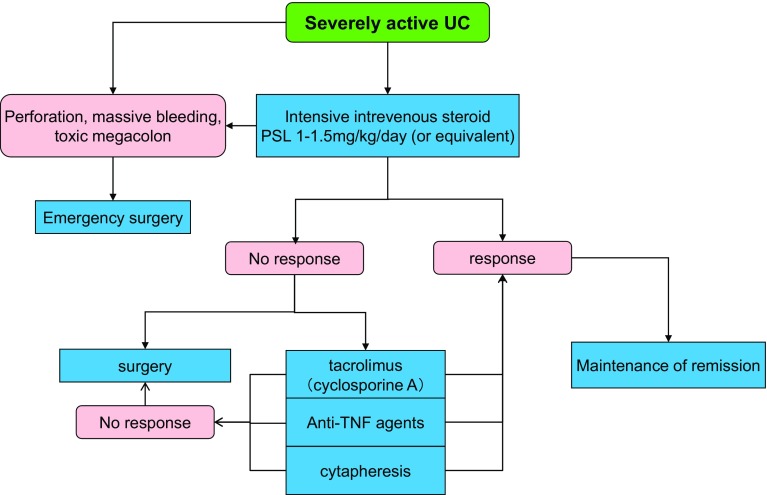
Remission induction treatment for severely active UC

***3) Treatment for severely active UC (Fig.*** ***5******)***

**CQ5-08. What are the indications of corticosteroids in the treatment of severely active UC?**

### Statements

- It is recommended to use steroids as first-line therapy (Recommendation grade: 1 (9), Evidence level: B).
- It is recommended to use PSL at a daily dose of 1–1.5 mg/kg (or equivalent) intravenously for severe UC (Recommendation grade: 1 (8), Evidence level: C).

### Comments

A systematic review in 2007 regarding the response to steroid therapy in severe UC included 32 clinical trials. It reported that the response rate of steroids in severe UC is 66% and approximately one-third of patients require colectomy within a short period of time (colectomy rate of severe patients = 404/1201, 34% (95% CI 31–36%)) [173].

There is only one study determining the optimal dose of steroids as first-line therapy for severe UC. Oral administration of steroids at 40 mg/day was as effective as 60 mg/day, but had less adverse events compared with 60 mg/day. Therefore, it is necessary to pay attention to side effects (infection, psychological complications, and thrombosis) when the high-dose steroid is used [174]. The ECCO Guidelines in 2012 stated that the dose of steroids for severe UC is generally 60 mg/day of methylprednisolone (equivalent to 80 mg/day of PSL) or hydrocortisone 100 mg four times daily [136].

It is reported that the response to steroids should be determined at least within the first 7 days when high-dose steroid is used for severe UC [174].

**CQ5-09. What are the indications of immunomodulators in the treatment of severely active UC?**

### Statements

- It is recommended to consider intravenous CyA for steroid-refractory severe UC (Recommendation grade: 2 (7), Evidence level: C).
- It is recommended to consider TAC for steroid-refractory severe UC (Recommendation grade: 1 (8), Evidence level: C).

### Comments

CyA blocks the translocation of the transcription factor, nuclear factor of activated T-cells (NFAT), to the nucleus by binding to calcineurin and, thereby, inhibits cytokine production. It is used mostly in acute severe UC and intravenously administered at 2–4 mg/kg continuously under total parenteral nutrition. Van Assche et al. reported that there was no difference between the initial doses of 2 mg/kg and 4 mg/kg in the therapeutic response at day 8 [175], suggesting that the optimal dose is 2 mg/kg. When treating with CyA, it is indispensable to monitor drug concentrations in the blood to maintain optimal drug levels and to avoid adverse events. Clinical improvement can be seen within a week or so after the initiation of treatment. The treatment period is usually up to 2 weeks since its long-term use increases the risk of side effects, including hypertension, seizures, sensory disturbance, hand tremor, gingival swelling, hirsutism, abnormal electrolytes, opportunistic infection, and renal dysfunction. The Cochrane review concluded that, although the therapeutic efficacy of CyA is confirmed for severe UC, evidence on it is limited [90].

TAC also inhibits calcineurin activity and thereby cytokine production [88].

TAC is used for steroid-dependent or -refractory UC. Blood trough levels for induction of remission are recommended to be 10–15 ng/ml [176, 177]. It is indispensable to monitor blood trough levels of TAC, similar to CyA, to maintain optimal drug levels and to avoid adverse events. Some patients complain of hand tremor and hot flushes during TAC therapy. The severity of headache varies from mild to very severe and may not improve even after lowering blood levels in some severe patients; therefore, attention should be paid. The incidence of renal dysfunction is reported at high blood concentrations but promptly recovers by lowering blood concentrations. It is necessary to establish evidence for the efficacy of TAC in severe UC [88]. In addition, the optimal trough level for severe UC is unknown. However, it is necessary to promptly raise blood trough levels for inducing remission in severe patients. The oral dose of TAC should be increased in a short time considering individual conditions. Attention should be paid to pneumocystis pneumonia and prophylactic use of sulfamethoxazole–trimethoprim should be considered in elderly patients.

**CQ5-10. What are the indications of anti-TNF agents in the treatment of severely active UC?**

### Statements

- Anti-TNF agents are recommended in patients who are refractory to conventional treatments (Recommendation grade: 1 (8), Evidence level: A).
- A comparative trial demonstrated that IFX is as effective as CyA in patients refractory to steroids (Evidence level: C).

### Comments

Large multicenter trials overseas indicated that anti-TNF agents are effective for induction and maintenance of remission in moderate-to-severe UC patients who are refractory to conventional treatments [104, 118, 171, 178–183]. The efficacy of IFX and ADA have been demonstrated in the ACT-1/ACT2 studies [118] and the ULTRA-1/ULTRA-2 studies [104, 182], respectively, but comparative studies between each type of anti-TNF agent have not been conducted. Moreover, the CYSIF study reported that IFX is comparable to CyA in terms of efficacy when they are administrated to steroid-refractory moderate-to-severe UC patients [91]. In addition, there are some reports which demonstrated that IFX is effective in avoiding surgery [181]. Since currently available comparative studies between CyA and IFX have variations in target blood levels of CyA, etc., further investigation is required to make a conclusion.

Anti-TNF agents are a recommended therapy for UC patients who are refractory to conventional treatments, although more evidence on the efficacy of anti-TNF agents is necessary.

**CQ5-11. What are the indications of CAP in the treatment of severely active UC?**

### Statements

- It is recommended that CAP should be considered as one of the treatments of choice to improve the remission rate because its steroid-sparing effect has been demonstrated in severely active UC patients (Recommendation grade: 1 (8), Evidence level: C).
- CAP is recommended to be conducted twice-weekly or more to induce more rapid remission (Recommendation grade: 1 (8), Evidence level: C).

### Comments

A meta-analysis reported that CAP is superior in terms of reducing steroids (OR 10.49, 95% CI 3.44–31.93), and improving response rates (OR 2.88, 95% CI 1.60–5.88) and remission rates (OR 2.04, 95% CI 1.36–3.07) in moderate-to-severe UC patients compared to conventional pharmacotherapy [130]. Furthermore, CAP causes severe adverse events less frequently compared to other drug treatments (OR 0.16, 95% CI 0.04–0.60) and is a safe therapeutic option [130]. However, the studies which the meta-analysis analyzed contained only a few RCTs; therefore, high-quality evidence that demonstrates the efficacy of CAP is still lacking.

A Japanese multicenter prospective RCT, although it was an open-label trial, reported that twice-weekly intensive treatment induced remission more rapidly (28.1 vs 14.9 days, *p* < 0.0001) and improved the remission rate (54.0 vs 71.2%, *p* = 0.029) compared with the weekly treatment [132].

Monotherapy with CAP is not common in severe UC patients, and CAP should be considered as a treatment of choice in combination with other treatments.

**Fig. 6:**
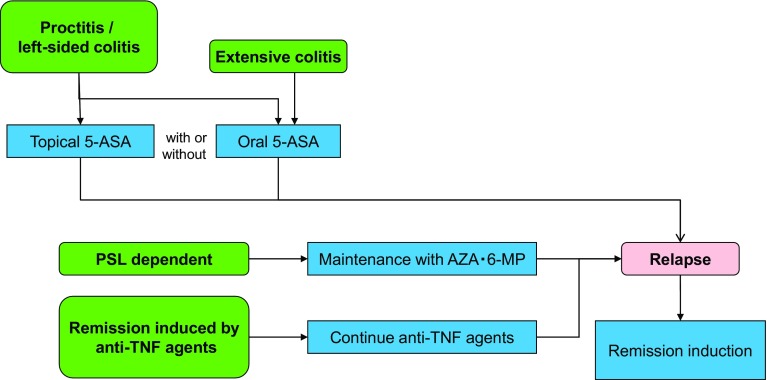
Maintenance treatment for UC in remission

***4) Maintenance treatment for UC in remission (Fig.*** ***6******)***

**CQ5-12. What are the indications of 5-ASA for UC in remission?**

### Statements

- Oral 5-ASA at a dose of 2 g/day or more is recommended to maintain clinical/endoscopic remission (Recommendation grade: 1 (9), Evidence level: A).
- 5-ASA enema is recommended for maintenance of remission in distal colitis (Recommendation grade: 1 (8), Evidence level: A).

### Comments

The efficacy of oral 5-ASA for maintenance of remission in UC has been analyzed by the Cochrane review, and it is effective for maintaining clinical as well as endoscopic remission [61]. High-dose 5-ASA is superior to low-dose in maintaining remission; therefore, it is advisable to use 2 g/day or more. In addition, SASP, a form of 5-ASA preparations, is equivalent to, or slightly more effective than 5-ASA for maintenance of remission, and its efficacy is dose-dependent. However, the incidence of its side effects or intolerant symptoms increases in a dose-dependent manner, therefore, a dose of 2–3 g/day is often chosen as a maintenance treatment.

There is no clear difference in efficacy for maintenance of remission between a once-daily administration and conventional 2–3 times a day administration [61]. Drug adherence between the two administration methods is also reported to be equivalent; however, this result was obtained in clinical trials. It is reported that multiple split dosing is associated with a lower adherence in daily clinical practice [184]. Lower adherence may lead to higher probability of relapse; therefore, once-daily dosing is more favorable to maximize the remission maintenance effect of this drug. Most of the results of dose comparison studies of individual 5-ASA preparations indicated that a higher dose of 5-ASA is unlikely to result in better maintenance effect; even in the study using time-dependent mesalazine, there was no difference in efficacy between 1.5 and 3.0 g/day [185].

Efficacy of topical 5-ASA (suppository and enema) in distal UC for maintenance of clinical and endoscopic remission has been confirmed by the Cochrane review and it is as effective as oral 5-ASA [59]. There are a few reports demonstrating that dose-increase of topical 5-ASA does not improve its remission maintenance effect. Mesalazine enema approved in Japan is effective and safe at a dose of 1 g once daily. There are a few reports suggesting the efficacy of combination treatment of oral 5-ASA with twice-a-week enema; however, further confirmatory studies are necessary since the number of patients included in these trials is limited [186, 187]. It is often difficult for patients to continue topical 5-ASA for a long time as a maintenance therapy; therefore, sufficient consideration should be given to patients’ acceptance.

**CQ5-13. What are the indications of immunomodulators for UC in remission?**

### Statements

- Immunomodulators (AZA/6-MP) are recommended in patients who are dependent on steroids or have difficulty in withdrawing steroids (Recommendation grade: 1 (9), Evidence level: A).

### Comments

It is recommended to use immunomodulators (AZA/6-MP) in patients who are dependent on or have difficulty in withdrawing steroids. Many studies demonstrated that continuing AZA after achieving remission provides remission rates of around 50% or higher. It is also known that, based on the results of multiple RCTs, immunomodulators are significantly effective in maintaining remission and tapering steroids [78, 188–191].

The recommended doses of immunomodulators are 1.5–2.5 mg/kg/day of AZA and 075–1.5 mg/kg/day of 6-MP; however, Japanese patients are known to be more likely to develop side effects because of their low metabolic capacity. 6-MP is not officially approved in Japan, therefore it is recommended to start with AZA at first and switch to 6-MP if the patient is intolerant to AZA. AZA is generally started at 25 mg/day and careful attention should be paid to both side effects and efficacy. In addition, there is a report suggesting that the effect of AZA monotherapy on maintenance of remission is equivalent to the combination therapy with 5-ASA and AZA, and there is no additive benefit of combined 5-ASA [191]; however, it is still common to use 5-ASA as a maintenance treatment along with AZA because experts’ evaluations of this study is not sufficient. It is also common to add AZA/6-MP when the patients cannot maintain remission by high-dose 5-ASA. Caution is required since the combination of 5-ASA and AZA inhibits AZA metabolism and may increase the incidence of side effects such as myelosuppression. Long-term tolerability is relatively good if the patient does not develop side effects in the initial several weeks, and the increase in the risk of malignancy, which was previously of concern, is reported as negative.

It is unclear whether or not CyA and Tac are effective for maintaining remission because of the absence of sufficient studies at present; therefore, AZA/6-MP is used as a maintenance therapy after achieving remission by CyA/TAC [88, 90].

**CQ5-14. What are the indications of anti-TNF agents for UC in remission?**

### Statements

- Long-term administration of anti-TNF agents is recommended as remission maintenance therapy for moderate-to-severe UC patients who achieved remission with anti-TNF agents (Recommendation grade: 1 (8), Evidence level: B).
- Maintenance of remission with anti-TNF agents provides higher likelihood of avoiding colectomy (Evidence level: B).

### Comments

The meta-analysis of 506 clinical trials concluded that anti-TNF agents are effective not only in inducing remission (relative risk (RR) 2.4, 95% CI 1.72–3.47) [183], but also in maintaining remission (RR 2.00, 95% CI 1.52–2.62) in UC patients. The long-term efficacy of maintenance therapy with IFX every 8 weeks for at least up to 52 weeks has been confirmed not only in CD patients, but also in UC patients that have been refractory to conventional therapy and achieve remission with IFX [118]. The probability of colectomy in patients with continued maintenance therapy with IFX is 9.5% within 54 weeks, which is significantly lower than 17% in patients with placebo (*p* = 0.02) [192]; therefore, the position statement of the World Organization of Gastroenterology stated that maintenance therapy with IFX should be considered in order to reduce the risk of colectomy [193].

On the contrary, even in the large-scale trials (ACT1/ACT2) which demonstrated the usefulness of IFX as a maintenance therapy, placebo-controlled re-randomization in order to examine its efficacy for maintenance of remission was not conducted [118] and, thereby, there is no evidence appropriately confirming its genuine efficacy for maintenance of remission. Therefore, the optimal length of treatment period after induction with IFX or its efficacy as a maintenance therapy after other induction treatments is not clear. The efficacy of long-term maintenance treatment with ADA after successful induction of remission with it is also confirmed as with IFX [104].

**CQ05-15. What are the indications of miscellaneous treatments for UC in remission and how are they used?**

### Statements

- Any other therapies than 5-ASA preparations, thiopurines, or anti-TNF agents are not clearly shown to have a remission maintenance effect and should not be used for maintenance of remission (Recommendation grade: 1 (8), Evidence level: C).

### Comments

A meta-analysis failed to demonstrate the non-inferiority of probiotics, which had been expected to have efficacy in maintenance of remission, compared to mesalazine in terms of relapse rates; 40.1% in the probiotic group and 34.1% in the mesalazine group (OR 1.33, 95% CI 0.94–1.90). It also failed to demonstrate the superiority of probiotics over placebo in terms of relapse rates within a year after induction of remission; 75% in the probiotics group and 92% in the placebo group (OR 0.27, 95% CI 0.03–2.68) [100].

Long-term use of TAC beyond 3 months for refractory UC patients following TAC-induced remission, and semi-monthly CAP following CAP-induced remission are expected as novel remission maintenance treatments; however, there is still no evidence on their efficacy in maintaining remission.

***5) Surgical treatment for UC***

**CQ5-16. What are the indications of surgery in UC?**

### Statements

- When patients are suffering from colonic perforation, massive bleeding, toxic megacolon, CRC or high-grade dysplasia, or severe disease which does not respond to medical treatments, surgery is recommended (absolute indications) (Recommendation grade: 1 (9), Evidence level: D).
- Surgery is also recommended for patients in whom adequate medical treatment is ineffective, or for patients whose daily life is impaired due to the disease, extraintestinal complications, or side effects of drugs (relative indications) (Recommendation grade: 1 (9), Evidence level: D).

### Comments

Because a delay in surgery increases the risk of postoperative complications, it is necessary to avoid a delay in the decision of surgery while monitoring response to medical treatments, side effects of the drugs, and complications, and consulting with specialists or surgeons if necessary [22, 137].

It is clearly necessary to conduct surgery in patients with life-threatening physiological conditions [22, 136]. Surgery is absolutely indicated for patients with a severe or fulminant disease who do not respond to sufficient medical therapies such as high-dose intravenous steroid, CAP, intravenous CyA, oral TAC, and anti-TNF agents [137].

Surgery should be considered at an appropriate timing because a delay in surgery increases the incidence of postoperative complications especially in patients with a severe disease or in elderly patients, who have low reserve capacity [194, 195].

It is difficult to uniformly determine relative indications of surgery because there is a wide variety in patients’ disease severity and conditions.

A relative indication of surgery is considered in patients who are suspected to have coexisting cancer because of the presence of stricture or low-grade dysplasia [137], but the surgical indication should be carefully determined in those patients because it is difficult to ascertain the presence of cancer [196].

To determine a surgical indication, gastroenterologists and surgeons should cooperate to fully explain issues associated with surgery to the patient and have enough discussion with the patient while considering his/her physical condition, social background, and preference [22, 136].

**CQ5-17. What are the surgical procedures for UC?**

### Statements

- It is recommended to perform total proctocolectomy with ileal pouch–anal anastomosis or ileal pouch–anal canal anastomosis as the standard surgical technique of elective surgery (Recommendation grade: 1 (9), Evidence level: D).

### Comments

The purpose of surgery is to remove the colon and rectum, the target organs of this disease; therefore, total proctocolectomy or subtotal colectomy is conducted in principle [22, 136].

Total colectomy with ileal pouch–anal (canal) anastomosis preserves the anus, providing satisfactory postoperative QOL; therefore, is often chosen as a standard surgical technique of elective surgery. Although the average number of daily bowel movements after surgery is 5–6 times, and patients may develop fecal incontinence, the rate of functional pouch (pouch functioning as a reservoir without ileostomy or resection) is high. Ileal pouch–anal anastomosis is highly curative while ileal pouch–anal canal anastomosis preserves good anal function and causes less fecal incontinence [138, 143, 197, 198].

Total (or subtotal) colectomy with ileostomy and mucous fistula of the sigmoid colon or Hartmann surgery may be performed in severe or emergent patients.

Total proctocolectomy with (permanent) ileostomy or subtotal proctocolectomy with ileorectal anastomosis may be performed considering anal function, age, and social background. Patients’ QOL after these procedures is also satisfactory [139, 140].

After surgical procedures with residual colorectal mucosa, it is necessary to pay attention to the development of cancer/dysplasia.

A systematic review overseas reported that laparoscopic surgery is not superior to open surgery in UC patients [199]. The judgment of surgeons is important in determining the indication of laparoscopic surgery and it is desirable to perform it at specialist centers that have sufficient experience [200].

It is necessary for the patient, the surgeon, and the physician to fully discuss to decide a surgical procedure, taking the patient’s physical and social conditions into account.

**CQ5-18. What are postoperative complications of UC and how are they treated?**

### Statements

- Anastomotic leak or bowel obstruction may develop postoperatively, and surgical treatments are recommended if necessary (Recommendation grade: 1 (9), Evidence level: D).
- It is recommended to use antibiotics for pouchitis (Recommendation grade: 1 (9), Evidence level: B).

### Comments

There is a possibility to develop infectious complications in patients under treatments with strong immunosuppressive effects. As patients who are administered steroids especially have an increased risk of anastomotic leak and infectious complications including wound infection, it is necessary to taper steroids during the preoperative period and be cautious about a choice of surgical procedure, and postoperative management [22, 137].

Pelvic sepsis including anastomotic leak occurs in 4–10% of patients as postoperative complications of total proctocolectomy with ileal pouch–anal anastomosis or ileal pouch–anal canal anastomosis, which is the standard operation for UC [143, 201]. There are other possible complications such as fistula, anastomotic stricture, pouch-related complications such as pouchitis [202]; however, the rate of functional pouch (pouch functioning as a reservoir without ileostomy or resection) is approximately 95% at 10 years [143, 201, 203]. The incidence of bowel obstruction is approximately 15% and surgical treatment may be necessary [143, 146, 201].

The incidence of pouchitis is reported to be approximately 30% in Western countries, which is higher than Japan. MNZ and CPFX are used for treating pouchitis, but the latter causes less side effects [99, 196]. There is no established treatment for patients who do not respond to antibiotics or who have repeated relapses and chronical progression.

In female patients, fertility may decrease after proctocolectomy with ileal pouch–anal (canal) anastomosis, but the course of pregnancy is benign until delivery. Defecation function may be affected by pregnancy, but recovers after delivery [147].

## 6. Treatments of CD

**Fig. 7:**
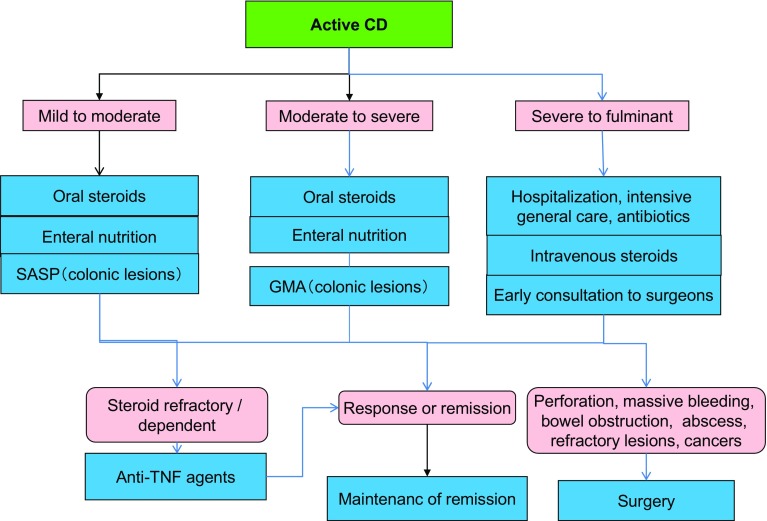
Remission induction treatment for active CD

***1) Treatment for mildly to moderately active CD (Fig.*** ***7******)***

**CQ6-01. What is the treatment of choice in mildly to moderately active CD?**

### Statements

- SASP or steroids are suggested for use in mildly to moderately active colonic CD (Recommendation grade: 2 (7), Evidence level: B).
- It is recommended to choose enteral nutrition or systemic steroids for the treatment of small intestinal lesions (Recommendation grade: 1 (8), Evidence level: B).
- Anti-TNF agents are recommended to be considered for steroid-dependent or -refractory patients (Recommendation grade: 1 (9), Evidence level: A).

### Comments

Multiple disease severity classifications of CD have been proposed and, in Japan, the Research Group for Intractable Inflammatory Bowel Disease proposed a severity classification which utilizes the CDAI and other indices (refer to CQ1–05). In daily clinical practice, severity is comprehensively assessed by patients’ subjective symptoms, and clinical and laboratory findings. The mild-to-moderate severity referred in this guideline means “patients who can attend outpatient clinics, can ingest orally, and do not have findings such as dehydration, fever, abdominal tenderness, bowel obstruction, or weight loss of 10% or more”.

Efficacy of SASP for mildly to moderately active CD is confirmed by a meta-analysis [62]. This meta-analysis demonstrated that SASP is effective only for colonic CD and is not as effective as steroids. Efficacy of mesalazine for active CD has been shown in several reports [63, 204–206], and it is commonly used and may be effective in clinical practice. However, a recent meta-analysis demonstrated that 5-ASA preparations are no more effective than placebo for induction of remission even at a high dose [62, 207].

Efficacy of steroids for induction of remission has been confirmed by meta-analyses [69, 70], and it was more effective when used longer than 15 weeks [70]. However, the optimal dose and period of treatment are undetermined. Side effects were observed more frequently in patients who received steroids compared to low-dose 5-ASA or placebo, but the withdrawal rates from the clinical trials due to side effects were not different from the placebo group or the low-dose 5-ASA group. In Europe and the US, budesonide, a steroid with fewer systemic side effects, is used for ileal and right-sided colonic lesions.

A meta-analysis comparing between enteral nutrition and steroids concluded that steroids are superior to enteral nutrition [121].

Anti-TNF agents can be a therapeutic option for moderately active CD patients, especially who are dependent on or refractory to steroids [103, 208, 209]. GMA is reported to be effective in patients with colonic CD who do not respond to conventional medical or nutritional treatments [133].

A meta-analysis studying the efficacy of antibiotics showed that they may be effective in inducing remission. However, many different types and combinations of antibiotics were included in the study; therefore, careful attention should be paid to interpret the data [94].

***2) Treatment for moderately to severely active CD (Fig.*** ***7******)***

**CQ6-02. What is the treatment of choice in moderately to severely active CD?**

### Statements

- Administration of oral steroids (PSL ≈ 40 mg/day) is recommended (Recommendation grade: 1 (8), Evidence level: A).
- It is suggested to consider enteral nutritional therapy, although its efficacy in induction of remission is comparable or slightly inferior to steroids (Recommendation grade: 2 (7), Evidence level: C).
- It is recommended to consider administrating anti-TNF agents to steroid-refractory patients (Recommendation grade: 1 (9), Evidence level: A).
- When patients with active colonic disease who do not respond to or intolerant to drug therapy or nutrition therapy, it is suggested to consider GMA (Recommendation grade: 2 (7), Evidence level: C).

### Comments

The moderate-to-severe severity referred in this guideline means, as defined overseas, “patients who have a CDAI score of 250–450 and do not respond to treatments usually administered to mild-to-moderate patients, or those who have a fever, remarkable weight loss, abdominal pain and/or abdominal tenderness, intermittent nausea/vomiting (without obstruction), or significant anemia” [210].

An RCT has demonstrated the efficacy of steroids for induction of remission in CD patients [70, 211]. However, steroids are not effective in maintaining remission [71]; therefore, a combination with immunomodulators such as AZA and 6-MP should be considered when the disease recurs during reducing steroids or shortly after steroid withdrawal, or repeatedly relapses after steroid withdrawal. In Western countries, methotrexate is used as an effective option when AZA or 6-MP is not tolerable due to side effects [212, 213].

The efficacy of anti-TNF agents in patients who do not respond to steroids or immunomodulators has been proved in both single-dose administration and scheduled repetitive administration [103, 214–219].

The use of GMA in CD patients with active colonic disease who do not respond to conventional drug therapy or nutritional therapy has been approved in 2010 in Japan [133].

***3) Treatment for severe*-*to*-*fulminant active CD (Fig.*** ***7******)***

**CQ6-03. What is the treatment of choice in severe-to-fulminant CD?**

### Statements

- It is recommended that patients should be usually hospitalized, be considered fasting, infusion, and blood transfusion, if necessary, and be administered antibiotics if they have symptoms suggestive of infection (Recommendation grade: 1 (9), Evidence level: D).
- It is recommended to administer steroids (PSL 40–60 mg/day) intravenously after excluding infections (Recommendation grade: 1 (8), Evidence level: A).
- It is recommended to consider administering anti-TNF agents to steroids-refractory patients (Recommendation grade: 1 (9), Evidence level: A).
- It is recommended to consult surgeons early when patients are in poor general condition and do not respond to anti-TNF agents (Recommendation grade: 1 (9), Evidence level: D).

### Comments

The severe-to-fulminant severity referred in this guideline means “patients who have persistent symptoms in spite of oral steroid administration or those who develop a high fever, persistent vomiting, bowel obstruction, rebound tenderness, cachexia, or abscess” [220].

Patients with severe-to-fulminant activity should usually be hospitalized and need intensive general care. Intravenous steroid administration should be preceded by oral administration because absorption of oral steroids is not stable and intravenous administration is superior in terms of pharmacokinetics [221]. The efficacy of steroids has been demonstrated by two placebo-controlled trials and six 5-ASA-controlled trials [70]. As many studies have demonstrated the efficacy of anti-TNF agents (IFX and ADA) in severe CD patients, these drugs can be considered as a treatment of choice if infectious complications such as abscess can be excluded or improved if exist [103, 214–219]. In addition, anti-TNF agents can be considered as a treatment of choice in fulminant CD patients although evidence on the efficacy of anti-TNF agents in such patients is limited. When treating CD patients who have unstable hemodynamics or peritoneal irritation symptoms, or those who do not respond to anti-TNF agents, it is desirable to consult surgeons for surgical indication early in the course.

***4) Perianal lesions***

**CQ6-04. What are the medical treatments for perianal lesions in CD?**

### Statements

- It is recommended to determine a surgical indication for perianal lesions properly based on an examination by experienced surgeons or proctologists and imaging investigations (Recommendation grade: 1 (9), Evidence level: D).
- It is suggested to use immunomodulators as a medical treatment for anal fistula (Recommendation grade: 2 (7), Evidence level: C).
- It is recommended to consider anti-TNF agents as a medical treatment for anal fistula if abscess is under control (Recommendation grade: 1 (8), Evidence level: A).

### Comments

The local pathology of perianal and anal canal diseases accompanying CD should be examined by experienced surgeons or proctologists, and investigation under anesthesia may be necessary [222, 223]. Imaging techniques including endoscopy, CT, MRI, and transanal US are useful in examining anal and rectal lesions, and it is important to assess the inflammatory changes in the anus and rectum with these examinations and properly determine the need for surgery [222, 223].

A meta-analysis indicated the efficacy of immunomodulators (e.g., AZA) for perianal fistulas [224]. Antibiotics such as MNZ are used in clinical practice, although there is only limited evidence on their therapeutic efficacy [225].

An RCT showed that IFX is effective for induction of remission of fistulas (90% of fistulas in this study were anal fistula) in CD patients [226]. Additionally, its efficacy for maintaining remission has also been confirmed [227]. A sub-analysis of the maintenance study of ADA demonstrated its efficacy for fistulas [217]. It is necessary to confirm that infection such as abscess has been controlled before administrating anti-TNF agents.

**Fig. 8:**
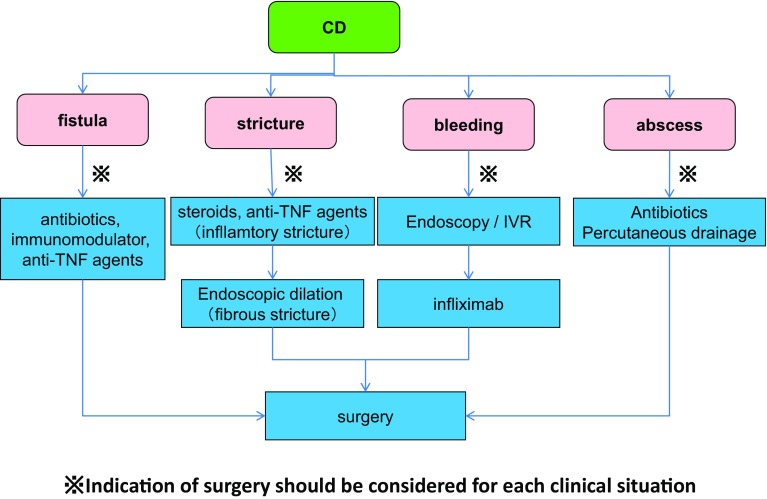
Treatment for intestinal complications of CD

***5) Intestinal complications (fistula, strictures, abscess, bleeding) (Fig.*** ***8******)***

**CQ6-05. Intestinal complications of CD (1): what is the treatment of choice for fistula?**

### Statements

- It is recommended that antibiotics, immunomodulators, and anti-TNF agents should be considered as drug therapies for perianal fistulas (Recommendation grade: 1 (9), Evidence level: C).
- It is recommended to consider anti-TNF agents for the treatment of enterocutaneous fistula without intestinal stenosis or complex fistula (Recommendation grade: 1 (8), Evidence level: D).
- Surgery is recommended for fistulas accompanied with abscesses, or internal fistulas accompanied with severe malabsorption (Recommendation grade: 1 (8), Evidence level: D).

### Comments

CD may be accompanied with internal fistulas such as entero–entero fistulas and external fistulas such as enterocutaneous fistulas. There is still no consensus regarding the necessity of treatments for asymptomatic fistulas [228].

Antibiotics, immunomodulators, and anti-TNF agents are used as medical treatments for fistulas.

1. There are few placebo-controlled studies about antibiotics therapy. However, it can be expected that MNZ or CPFX improves symptoms and closes fistulas [229–231].
2. Efficacy of immunomodulators has been proved by a meta-analysis of RCTs overseas (OR 3.09, 95% CI 2.45–3.91) [224].
3. Regarding anti-TNF agents, IFX, ADA, and certolizumab are effective for 50% or more reduction of fistulas in number, or complete closure of fistulas in patients with maintenance therapy with these drugs. However, the efficacy has not been confirmed in a short term (4–18 weeks) [218]. Moreover, it has been reported that a combination with antibiotics and anti-TNF agents increases the therapeutic efficacy for anal fistula [232].
4. It is reported that anti-TNF agents are effective in one-third of CD patients with enterocutaneous fistula (in the absence of intestinal stenosis or complex fistula). On the other hand, the efficacy of anti-TNF agents for internal fistula is low. [233].

Surgery should be considered when medical treatments do not improve fistulas. Internal fistulas accompanied with severe malabsorption, repetitive urinary tract infection, an excess leak of enteric juice from cutaneous fistula, perianal pain, or abscess formation are indications for surgery.

**CQ6-06. Intestinal complications of CD (2): what is the treatment of choice for strictures?**

### Statements

- Short-term steroid administration or anti-TNF agents are recommended for inflammatory strictures (Recommendation grade: 1 (8), Evidence level: D).
- Endoscopic dilation or surgery is recommended when the obstructive symptoms do not improve by drug therapy alone (Recommendation grade: 1 (8), Evidence level: C).

### Comments

Intestinal strictures include those caused by mucosal edema accompanied with inflammation or those caused by intestinal fibrosis. Strictures that are mainly caused by inflammation may be improved with anti-inflammatory therapy such as steroids [234]. When anti-inflammatory therapy does not improve symptoms, intestinal fibrosis should be suspected, and the indication of endoscopic dilation should be considered based on the length and number of the strictures and the presence of ulcers.

The indication of endoscopic dilation should fulfill the following: (1) stricture length is 5 cm or less and its curve is not severe; (2) fistula and abscess related to stricture are absent; (3) deep ulcers are absent in the stricture. Especially when patients do not fulfill (2), surgery should be considered.

A meta-analysis has confirmed that therapeutic effects are observed in 58% of CD patients who underwent endoscopic dilatation (average observation period is 33 months). It is reported that surgery can be avoided by endoscopic dilatation in patients who have stricture less than 4 cm in length [235].

Furthermore, it has been reported in Japan that a combined use of dilation with immunomodulators or biologics may be effective for avoiding surgery in CD patients [236, 237].

**CQ6-07. Intestinal complications of CD (3): what is the treatment of choice for bleeding?**

### Statements

- Attempt to hemostasis by endoscopy or interventional radiology is recommended, along with general care (Recommendation grade: 1 (8), Evidence level: D).
- IFX is recommended as a drug therapy (Recommendation grade: 1 (8), Evidence level: D).
- Surgical treatment is recommended if hemostasis cannot be achieved by the conservative therapy (Recommendation grade: 1 (9), Evidence level: D).

CD causes severe bleeding on rare occasions. Medical therapy including prohibiting food intake and administrating intravenous fluid should be aggressively started and bowel rest should be attempted. There are a few reports suggesting that steroids or IFX were an effective medical therapy for hemostasis [238, 239]. In addition, another report indicated that immunomodulators reduced the risk of lower gastrointestinal bleeding [240]. If possible, endoscopic hemostasis should be attempted. Angiography with intra-arterial vasopressin or transcatheter arterial embolization was reported to be effective [241, 242], but arterial embolization may cause intestinal necrosis. Surgical treatment is necessary if the medical therapy is not effective for hemostasis. It is reported that surgery is required in 20–90% for the first bleeding and 30–35% for the recurrent bleeding after conservative treatment [243, 244].

**CQ6-08. Intestinal complications of CD (4): what is the treatment of choice for abscess?**

### Statements

- Antibiotics administration, cutaneous drainage, and/or incision drainage are recommended after imaging examinations such as CT, US, and MRI (Recommendation grade: 1 (9), Evidence level: D).
- Administration of anti-TNF agents after treating abscess reduces the risk of recurrence of abscess (Evidence level: D).

### Comments

Transmural inflammation of CD may cause abdominal abscesses and its incidence in Japan is reported to be approximately 10% [245]. CT, MRI, and US examinations are useful in diagnosing abscess [246]. Medical management with fasting and infusion is basically considered. In addition, administration of broad-spectrum antibiotics should be considered and percutaneous drainage, if possible, should be conducted. Draining methods include CT-guided or ultrasound-guided percutaneous drainage and surgical drainage with small incision [247, 248]. In patients whose abscess is not improved or recurs after percutaneous drainage, surgery is necessary.

In patients who are accompanied with abscesses, it is necessary to identify the intestine responsible for the abscess formation and consider a surgery to resect the affected bowel.

The results of ACCENT II indicated that IFX is not associated with recurrence of abscess [249], and there are case reports suggesting that anti-TNF agents after abscess drainage are effective to prevent recurrence. Therefore, treatment with anti-TNF agents may be considered after appropriate drainage of abscess, although surgery is generally necessary especially for intractable patients with recurring abscess formation.

***6) Other gastrointestinal lesions***

**CQ6-09. What is the treatment of choice for upper gastrointestinal involvements in CD?**

### Statements

- Although evidence is scarce, it is suggested to consider proton pump inhibitors (PPI), steroids, thiopurines, and IFX as treatments for active upper gastrointestinal tract lesions (Recommendation grade: 2 (7), Evidence level: D).
- It is recommended to administer steroids and/or thiopurine for edematous stenosis, and consider endoscopic balloon dilation or surgery (gastrojejunostomy, or strictureplasty) for fibrous strictures (Recommendation grade: 1 (8), Evidence level: D).

### Comments

Even though evidence on treatment for upper gastrointestinal lesions in CD is scarce, there are some reports suggesting that PPI, steroids, thiopurines [250, 251] or IFX [252] were effective for active upper gastrointestinal lesions. In addition, there is a Japanese report about the efficacy of crushed 5-ASA powder preparation [253].

Endoscopic balloon dilation may be effective for a single and short gastric or duodenal stricture [254]. When endoscopic balloon dilation is difficult to conduct or is ineffective, surgery (gastrojejunostomy, or strictureplasty) should be considered [250, 251, 255].

A review based on experts’ opinions recommended that PPI is the first choice of treatment for upper gastrointestinal lesions of CD without strictures, and then treatments with steroids, thiopurines, and IFX follow. Endoscopic balloon dilation is recommended as first-line therapy for patients with strictures, and then PPI, steroids, thiopurines, and surgery follow [256].

It has been reported that the presence of upper gastrointestinal lesions of CD is a prognostic factor for disease progression over time [257].

**Fig. 9:**
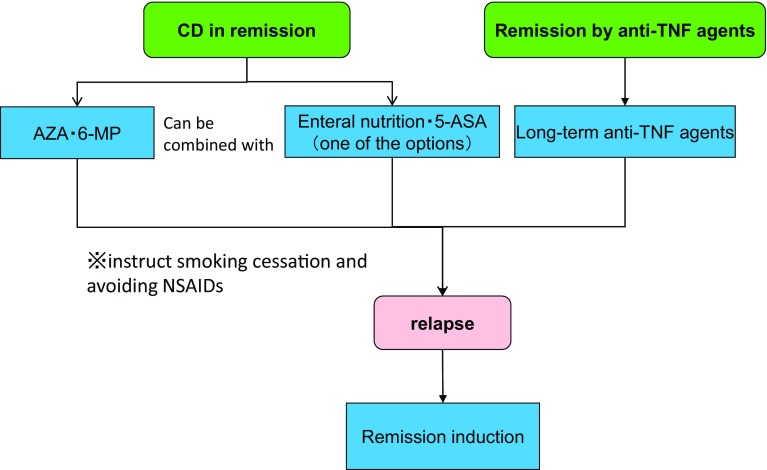
Maintenance treatment for CD in remission

***7) Maintenance treatment for CD in remission (Fig.*** ***9******)***

**CQ6-10. What are the things to do in daily life to help prevent recurrence of CD in remission?**

### Statements

- It is recommended to instruct smokers to quit smoking (Recommendation grade: 1 (9), Evidence level: B).
- It is recommended to avoid prolonged use of NSAIDs (Recommendation grade: 1 (8), Evidence level: C).

### Comments

It has been demonstrated that smoking is related to refractoriness or recurrence of CD [258], and smoking cessation can improve refractoriness and reduce the rate of recurrence [55]. The meta-analysis in 2006 that included 9 studies indicates that the OR of the risk of smoking for worsening CD is 1.76 (95% CI 1.40–2.22) [6]. Patients with CD should refrain from frequent or excessive drinking because alcohol may affect the function of the intestinal tract. Additionally, it is considered that unbalanced dietary habits can become a risk factor for recurrence of CD because nutrition therapy is effective for CD patients.

An association between psychological stress and recurrence of CD has been reported [259]. It is important for patients with CD to avoid stress as much as possible or try not to build up stress. As NSAIDs are known to cause gastrointestinal damage as well as become a recurrence or worsening factor of CD, it should be avoided to take NSAIDs as much as possible and, if analgesics or antipyretics are needed, it is desirable to take acetaminophen or COX-2 inhibitors for a short period [2, 9].

**CQ6-11. What is the treatment of choice to prevent recurrence of CD in remission?**

### Statements

- It is recommended to administer thiopurine (AZA/6-MP) to maintain remission (Recommendation grade: 1 (9), Evidence level: B).
- Scheduled maintenance administration of anti-TNF agents is recommended when anti-TNF agents induced remission (Recommendation grade: 1 (9), Evidence level: B).
- It is recommended that enteral nutrition therapy and 5-ASA should be considered as therapeutic options for maintenance of remission (Recommendation grade: 1 (8), Evidence level: C).

### Comments

1. Thiopurine

It has been reported that AZA and 6-MP are effective for reduction or discontinuation of steroids during the remission maintenance period, and for long-term maintenance of remission. The optimal dose of AZA is typically 1.0–2.5 mg/kg/day, whereas the dose of 6-MP is approximately half of AZA, but high-dose is considered to be superior to low-dose in terms of efficacy (OR for maintenance of remission with AZA 2.32, 95% CI 1.44–3.49 and OR for maintenance of remission with 6-MP 3.32, 95% CI 1.40–7.87) [260, 261]. However, it is necessary to consider the following: AZA and 6-MP may develop severe side effects; the optimal dose for efficacy or the threshold for side effects varies widely among individuals; as the recommended doses of AZA/6-MP are intended for westerners, even a lower dose of AZA/6-MP may still be effective in Japanese patients but can also cause side effects. A meta-analysis reviewed 3 studies examining the outcome of discontinuation of AZA in 163 patients who maintained remission with thiopurines. The result demonstrated that continuation of AZA is effective to prevent from recurrence of CD [81]. Based on the report that showed the efficacy of continuous administration of AZA beyond 2 years, it seems that administrating AZA for 3–4 years is practical as far as patients maintain remission without side effects (RR of relapse with AZA 0.39, 95% CI 0.21–0.74) [77]. A meta-analysis in 2009 reported that postoperative administration of thiopurines can reduce endoscopic recurrence [81].

2. Anti-TNF agents

It has been demonstrated that anti-TNF agents are effective in preventing recurrence in luminal CD patients compared to placebo, according to the meta-analysis in 2011 which included 5 clinical trials (including 1390 CD patients) (RR of relapse 0.71, 95% CI 0.65–0.76) [103]. Therefore, IFX at a dose of 5 or 10 mg/kg every 8 weeks, ADA at a dose of 40 mg every week (unapproved in Japan) or every other week, and certolizumab at a dose of 400 mg every 4 weeks are effective for maintaining remission in CD patients who have responded to each biological drug. However, data on safety profile related to long-term administration is not yet sufficient.

3. Enteral nutrition therapy

Enteral nutrition therapy has a good safety profile as a long-term remission maintenance treatment, but home total enteral nutrition therapy is difficult to continue for a long term because of poor tolerance and low convenience. Partial enteral nutrition therapy can be expected to improve tolerance and convenience, and patients can enjoy eating. There are only two RCTs to compare between enteral nutrition and placebo (no eating restrictions). However, it is reported that when patients intake 30–50% of calories by enteral nutrition, the efficacy for maintaining remission is greater compared to when they only take normal diet [125, 127, 262]. Even though there is no evidence on the efficacy of enteral nutrition that continues beyond a year, if the patient has no problem in tolerability and convenience, it seems to be desirable to continue the therapy as long as possible.

4. 5-ASA preparations

A review in 2010 reported that 5-ASA is no more effective for maintaining remission after inducing remission by medical therapy compared to placebo [260]. On the other hand, the Cochrane review in 2011 that analyzed 9 RCTs indicated that the efficacy of 5-ASA in patients in remission after surgery is slightly superior to placebo in terms of risk reduction of recurrence [65]. However, careful interpretation is necessary for the results.

5. Home parenteral nutrition

Sufficient digestion and absorption can no longer be expected from the intestinal tract when the residual small intestine becomes 1 m or less due to extensive small intestinal resection because of stricture or perforation in CD patients with small intestinal lesions, or small intestinal resection with frequent surgeries. Therefore, a central venous catheter should be placed in order to provide necessary nutrition and to enable patients or family members to maintain the intravenous management at home [263].

***8) Surgical treatment for CD***

**CQ6-12. What are the indications of surgery in CD?**

### Statements

- Surgery is recommended for perforation, massive bleeding, cancer, bowel obstruction refractory to medical therapy, and abscess (absolute indications) (Recommendation grade: 1 (9), Evidence level: D).
- Surgery is recommended for refractory stenosis, internal fistula, external fistula, refractoriness to medical treatment, refractory extraintestinal complications (e.g., growth retardation, pyoderma gangrenosum), and refractory perianal lesions (relative indications) (Recommendation grade: 1 (9), Evidence level: D).

### Comments

Although there has been no new evidence to revise the statements in this section since the last version of the guideline was published, many physicians and surgeons empirically agree with these statements. Endoscopic balloon dilation may be occasionally chosen for stenosis rather than surgery; however, there are some limitations for the indication of endoscopic balloon dilatation and it should be conducted under backup by surgeons [264]. Although abscess is considered to be eventually an indication for surgery [265], emergency surgery may be avoided with administration of antibiotics and percutaneous drainage.

**CQ6-13. What are the surgical procedures for CD?**

### Statements

- For the treatment of stricture or fistula formation, resection of the affected intestine, or strictureplasty for the former, if possible, is recommended (Recommendation grade: 1 (9), Evidence level: D).
- Perianal lesions refractory to medical therapy are recommended to be treated by local procedures such as seton drainage, stoma formation, or rectal amputation (Recommendation grade: 1 (9), Evidence level: D).

### Comments

Patients with CD often develop postoperative recurrence and may require multiple repeated surgeries. Since the length of the unaffected intestine of the resected margin does not have an impact on the recurrence [266], resection of the intestine should be limited to the affected lesion that is responsible for symptoms not improved by medical treatment. In addition, surgical management of the healthy intestine and other organs affected by adjacent fistula or inflammation, or drainage of abscess are sometimes required.

Strictureplasty such as Heineke–Mikulicz, Finney, and Jabouley methods are performed for strictures. Strictureplasty is utilized for the purpose of preserving as much unaffected intestine as possible because the postoperative recurrence rate is not different from intestinal resection [267].

Among different types of anastomoses (end-to-end, end-to-side, side-to-side, functional end-to-end), a report demonstrated that functional end-to-end anastomosis has a lower incidence of anastomotic leak and a longer time to recurrence compared with end-to-end anastomosis [268], but another report showed the opposite results [269], and thus no consensus has been made.

Laparoscopic surgery has been reported to be superior to or comparable to open surgery in terms of cosmetic outcome, recovery of bowel motility, and the length of hospitalization [270, 271]. It is advisable that the indication of laparoscopic surgery for patients who will undergo re-surgery or those with abscess or fistula formation should be carefully discussed and the surgery is desirable to be performed at specialist centers [208].

Drug therapies such as MNZ or CPFX should be used for intractable perianal lesions in addition to the medical therapy for the intestinal lesions. Perianal fistulas are the most common form of perianal lesions and, if perianal fistulas are refractory to drug therapies, seton drainage is performed. Some patients may further need immunomodulators and/or anti-TNF agents, or surgical procedures such as stoma formation and/or rectal amputation [223].

In Japan, there are many reports of anorectal cancer and its incidence is recently increasing; therefore, careful attention should be paid to anorectal cancer when treating anorectal lesions [272].

**CQ6-14. What are postoperative complications of CD and how are they treated?**

### Statements

- Short-term postoperative complications include anastomotic leak, abdominal abscess, and bowel obstruction and these are recommended to be treated conservatively or surgically (Recommendation grade: 1 (9), Evidence level: D).
- Long-term postoperative complications include small intestinal failure, and it is recommended to be treated with nutrition therapy (Recommendation grade: 1 (8), Evidence level: D).

### Comments

Patients in whom residual small intestine becomes less than 150 cm as a result of intestinal resection may be unable to absorb water, electrolytes, and nutrients and may require supplementation by parenteral nutrition or home parenteral nutrition [150].

Intestinal lesions of CD may recur early after surgery, and the rate of endoscopic recurrence around the ileocolonic anastomosis after ileocecal resection reaches as high as 72% within a year [273]. The re-operation rates are reported to be 16–43% at 5 years and 26–65% at 10 years [274, 275].

The appearance of lesions (morphological recurrence) precedes clinical relapse when post-operative recurrence occurs; therefore, imaging examinations such as endoscopy are useful to diagnose it [208].

The Cochrane review states on prevention of postoperative recurrence as follows: MNZ is more effective but causes more side effects than placebo; mesalazine and AZA/6-MP are effective for prevention of clinical recurrence and endoscopic severe recurrence; the rate of endoscopic recurrence is higher in mesalazine than AZA/6-MP, but mesalazine causes fewer side effects; IFX or budesonide does not have sufficient data showing efficacy [276].

As stated above, there is no established standard for prevention of postoperative recurrence, and therefore it is necessary to decide the strategy to prevent postoperative recurrence in consideration of the subject, the start time, medication, side effects, tolerability, cost, etc.

## 7. Extraintestinal complications

**CQ7-01. What are extraintestinal complications of IBD and how are they treated?**

### Statements

- There are two types of extraintestinal complications of IBD; those associated with the activity of intestinal lesions; e.g., a group of peripheral arthritis, erythema nodosum, episcleritis, oral aphtha, etc.; those unassociated with the activity of intestinal lesions; e.g., pyoderma gangrenosum, uveitis, sacrum arthritis, ankylosing spondylitis, primary sclerosing cholangitis (PSC), etc.
- It is recommended to treat intestinal inflammation first when intestinal lesions are active (Recommendation grade: 1 (8), Evidence level: D).
- Local or systemic steroids are recommended as first-line therapy for pyoderma gangrenosum (Recommendation grade: 1 (8), Evidence level: D).
- It is suggested to consider the administration of anti-TNF agents for extraintestinal complications (Recommendation grade: 2 (7), Evidence level: B).

### Comments

There are two types of extraintestinal complications of IBD; those associated with the activity of intestinal lesions (a group of peripheral arthritis, erythema nodosum, episcleritis, oral aphtha, etc.) and those unassociated with the activity of intestinal lesions (pyoderma gangrenosum, uveitis, sacrum arthritis, ankylosing spondylitis, PSC, etc.). It is necessary to actively control inflammation of intestinal lesions in either case.

5-ASA preparations such as SASP are first-line therapy for arthritis. NSAIDs should not be used because they may worsen intestinal lesions, but a short-term administration of COX-2 inhibitors is relatively safe [277, 278]. Local or systemic steroids are used to treat pyoderma gangrenosum [279, 280]. Several case reports indicated that CAP was effective in patients with pyoderma gangrenosum refractory to steroids [281–283]. Among eye lesions, uveitis may cause blurring of vision; therefore, a consultation of ophthalmologists is warranted when uveitis is suspected [223].

An RCT and a non-RCT that included IBD and non-IBD patients reported that IFX was effective for pyoderma gangrenosum, arthritis, uveitis, ankylosing spondylitis, etc. [284, 285].

PSC is more frequently accompanied with UC than CD, and increases the risk of cholangiocarcinoma and CRC.

## 8. Cancer surveillance

**CQ8-01. How should screening and surveillance for UC-associated cancers be conducted?**

### Statements

- Screening colonoscopy is recommended 8 years after the disease onset (Recommendation grade: 1 (8), Evidence level: C).
- Patients with left-sided colitis or extensive colitis are recommended to undergo surveillance colonoscopy annually or biennially after the screening colonoscopy (Recommendation grade: 1 (8), Evidence level: C).
- Target biopsy with chromoendoscopy is recommended rather than random biopsy during colonoscopy (Recommendation grade: 1 (8), Evidence level: B).

### Comments

The incidence of CRC in UC patients is significantly higher compared to the general population. According to the meta-analysis including population-based cohort studies, the standardized incidence ratio of CRC among UC patients was 2.39 (95% CI 2.1–2.73) and the standardized incidence ratio of CRC in the patients with extensive colitis was 4.8 (95% CI 3.9–5.9) [286]. The American Gastroenterology Association (AGA) technical review on the diagnosis and management of colorectal neoplasia in IBD in 2010 [287] recommended that all UC patients should undergo screening colonoscopy 8 years after the disease onset, and, thereafter, patients with left-sided colitis or extensive colitis are recommended to have surveillance colonoscopy annually or biennially. The patients whose disease onset was unclear are recommended to have screening colonoscopy as those with more than eight years’ disease duration. Furthermore, when the patient is diagnosed with PSC, surveillance colonoscopy is recommended annually after the diagnosis of PSC. According to cross-over studies and the meta-analysis including RCTs, the use of chromoendoscopy with 0.1% of indigo carmine [288] or 0.1% of methylene blue [289] showed significantly higher intraepithelial neoplasia detection rate than white light endoscopy. In the US, biopsy from the areas with suspected malignant lesion in addition to 4 random biopsies every 10 cm is recommended during surveillance with white-light colonoscopy in patients with long-standing UC.

**CQ8-02. How should screening and surveillance colonoscopy for CD-associated cancers be conducted?**

### Statements

- It is proposed that CD patients have screening colonoscopy 8 years after the disease onset, and thereafter the patients whose disease affects more than one-third of the large intestine undergo surveillance colonoscopy annually or biennially (Recommendation grade: 2 (7), Evidence level: D).

### Comments

The risk of CRC in CD patients is slightly increased but the risk of small intestinal cancer is substantially high. According to the Minnesota population-based study, the standardized incidence ratio of CRC and small intestinal cancer was 1.9 (95% CI 0.7–4.1), and 41.1 (95% CI 8.5–120), respectively, among CD patients [290]. The incidence of small intestinal cancer is remarkably higher in CD patients than the general population. On the other hand, a study involving 770 Japanese CD patients showed that the standardized incidence ratio of CRC (including anal canal cancer) is 3.23 (95% CI 1.28–5.29), which is significantly high [291]. The AGA technical review in 2010 recommended that all CD patients should undergo screening colonoscopy 8 years after the disease onset, and, thereafter, the patients whose disease affects more than one-third of the large intestine should undergo surveillance colonoscopy annually or biennially [287]. The longitudinal study involving 259 CD patients with a disease duration of 8 years or more whose disease affects more than one-third of the large intestine demonstrated that the detection rate of dysplasia or colon cancer at the 4th surveillance colonoscopy (the median time from the index colonoscopy was 7.2 years) was 22% [292]. As a method of screening or surveillance colonoscopy, it remains undetermined whether 4 random biopsies every 10 cm should be obtained in addition to biopsies from the area of suspected neoplastic lesion. On the other hand, in Japan, it is reported that the complication of rectal and anal canal cancers are more prevalent, different from Western countries. Therefore, annual surveillance with a digital rectal examination, endoscopic biopsy, brushing cytology, tumor markers (CEA, CA19-9), and pelvic CT/MRI is recommended for CD patients who have been suffering from rectal and perianal lesions (ulcer, stricture, or fistula) for more than 10 years [293].

## 9. IBD in special situations

**CQ9-01. How should pregnancy and delivery be managed in IBD patients?**

### Statements

- It is recommended to choose treatment during pregnancy or lactation in IBD patients through sufficient discussion between physicians and patients in consideration of risks and benefits of each individual patient (Recommendation grade: 1 (9), Evidence level: C).
- It is generally recommended to continue treatment during pregnancy in IBD patients since benefits of treatment exceed risks of drugs in most patients (Recommendation grade: 1 (9), Evidence level: B).

### Comments

It is an important issue how to treat IBD patients during pregnancy and lactation in order to achieve safe delivery and lactation because IBD tends to develop among young people. The attending physicians should cooperate with obstetricians and pediatricians to manage IBD patients to make childbirth safely. The attending physicians should understand that pregnancy is a sensitive issue in which complications (spontaneous abortion, congenital anomaly, etc.) can occur with a certain probability, and should explain the risks of the complications to the patients (baseline risks are 15% for spontaneous abortion, 10% for infertility, and 3–5% for congenital anomaly [294]).

Fertility of female IBD patients in remission is the same as healthy individuals. The infertility rate is increased in active CD patients. Although the rate of infertility triples and increases to 48% in female UC patients after total colectomy with ileoanal anastomosis [295], it is possible for the patients to be pregnant by artificial insemination. Male patients who are taking SASP have reduced fertility, but the ability returns to normal after discontinuing the drug [296].

The risks of giving birth prematurely and having low-birth-weight infants are slightly increased in pregnancy of patients with active IBD, but patients can generally have a normal pregnancy and safe delivery as long as remission is maintained [297–303].

Data on pregnancies of IBD patients have been recently collected overseas. The mainstream opinion on this issue follows: the most serious risk to mothers and fetuses during pregnancy is the disease activity of IBD; therefore patients should continue treatment because the benefits of treatment generally exceed the risks caused by treatment [299–301]. Although data on Japanese patients are scarce yet, similar outcomes to overseas have been reported [302, 304].

The medical package inserts of most drugs in Japan state that administration should be avoided during pregnancy and lactation; however, none of the standard medications used for IBD in Japan are included in the list of drugs that should be carefully used during pregnancy and lactation described in the guidelines of the Japanese Society of Obstetrics and Gynecology [294].

1. Medication for pregnant women

Methotrexate must be avoided during pregnancy because there is evidence of its teratogenicity [305]. Care must be taken about over-intake of vitamin A during nutrition therapy [305] (the upper limit of retinol from 3 months before pregnancy to the first 3 months: 3000 μgRE, one packet of Elental^®^: 216 μgRE). SASP has an anti-folic activity and its administration is considered to be a high-risk factor for neural tube defects. Although there is no evidence to prove the preventive effect of folic acid supplementation, administrating folic acid at a daily dose of 4–5 mg before pregnancy beyond the first trimester is advisable (a folic acid 5 mg tablet (Foliamin^®^) is available in Japan) [294]. Even though the administration of AZA, CyA or TAC to pregnant women is written as “contraindicated” in their data sheets, it has not been proved that they have clinically significant teratogenicity or fetal toxicity [300, 305, 306]. When female patients receiving these drugs become pregnant, it is important to re-evaluate whether or not the treatment is necessary at this point; the treatment is discontinued if it is judged it can be discontinued; the treatment should be continued after explaining the fetal risk if it is desirable to continue [294]. Since IFX and ADA actively cross the placenta and transfer to the fetus from the late second trimester, discontinuing these drugs should be considered during the second trimester if possible [299, 307].

2. Administration during lactation

Breastfeeding should not be stopped based on misinformation since it reduces the risks of infection and mortality in infants [294]. 5-ASA, SASP, PSL, and anti-TNF agents are safe during lactation although most drugs are secreted into breast milk, to some extent [294, 305, 308, 309]. MNZ, CPFX, CyA, TAC, and methotrexate are transferred from milk to infants, therefore they should be avoided during lactation as much as possible [294, 300, 305, 308].

Infants born to mother receiving IFX or ADA are in an immunosuppressed state. BGC and live vaccine should be avoided until 6 months old [309, 310].

There is no evidence of absolute safety of drug administration during pregnancy and lactation, and new information is always being added; therefore, the attending doctor should make an effort to access the latest information, collaborating with the obstetrician. Data in Japan Drug Information Institute in Pregnancy provided by National Center for Child Health and Development (http://www.ncchd.go.jp/kusuri/index.html) are most updated and useful. Patients can also have an access to the data.

**CQ9-02. How should elderly IBD patients be managed?**

### Statements

- Treatments for elderly IBD patients are mostly the same as those for non-elderly patients; however, it is recommended to determine the appropriate timing of surgery in elderly patients with severe activity, keeping in mind that a delay in diagnosis and/or surgery may result in the life-threatening prognosis (Recommendation grade: 1 (9), Evidence level: C).
- When treating elderly patients who are refractory to one or more immunosuppressive therapies, it is recommended to consult specialists without delay (Recommendation grade: 1 (9), Evidence level: C).

### Comments

Many reports demonstrated that there is no significant difference between elderly and young patients in terms of severity and clinical course of the disease, therefore treatment strategy in elderly patients is mostly the same as young patients [311, 312]. However, attention should be paid to side effects and drug–drug interactions because elderly patients have poor organ reserve capacity and comorbidities, and receive multiple medications [311].

Since there are many diseases that must be ruled out such as infectious enteritis including intestinal tuberculosis, drug-induced enteritis, and ischemic enteritis in elderly IBD patients, the diagnosis is sometimes delayed [313]. Elderly patients are more prone to deterioration of nutritional status, or reduced activities of daily living due to restriction of daily living compared to young patients [311, 314]. Complications such as venous thrombosis and infection may especially affect the life prognosis [311].

Infectious diseases caused by immunosuppressive therapies (cytomegalovirus infectious disease and pneumocystis pneumonia), steroid-induced side effects including reduction of bone mineral density, hyperglycemia, adrenal insufficiency, and psychological symptoms, nephropathy caused by calcineurin inhibitors, and cardiac failure caused by anti-TNF agents are more likely to occur in the elderly compared to the young [311, 314].

The indication of anti-TNF agents for elderly IBD patients is similar to that for young IBD patients, but there is a report that the response to the therapy is lower and the risk of serious infection and death is higher in elderly patients than young patients [315]. In addition, sufficient examinations are necessary before starting anti-TNF agents because the risk of latent tuberculosis is also higher in the elderly patients [311].

Elderly IBD patients are more likely to develop complications such as massive bleeding and toxic megacolon due to a delay in surgery. Moreover, it has been reported that the rate of perioperative death was high due to postoperative complications such as pneumonia [316]. Therefore, if one immunosuppressive therapy is insufficiently effective for elderly IBD patients, it is recommended to consult specialists considering the early indication of surgery [311].

The surgical procedure for elderly UC patients should be performed in the same way as young patients as far as the anal sphincter muscle function is preserved since ileal-pouch anal (canal) anastomosis is not contraindicated even in elderly patients. However, permanent stoma formation may be selected considering patient’s anal sphincter muscle function or ileorectal anastomosis may be selected considering patient’s QOL [311].

## Appendix

The members of the Guidelines Committee who created and evaluated the JSGE “Evidence-based clinical practice guidelines for inflammatory bowel disease in Japan” are listed below.

### Creation Committee

Chair: Fumiaki Ueno (Ofuna Central Hospital).

Vice-Chair: Toshiaki Watanabe (Department of Surgical Oncology, University of Tokyo).

Members: Nagamu Inoue (Center for Preventive Medicine, Keio University School of Medicine), Fumio Omata (Gastroenterology Division, Department of Internal Medicine, St.Luke’s International Hospital), Jun Kato(Second Department of Internal Medicine, Wakayama Medical University), Reiko Kunisaki (Inflammatory Bowel Disease Center, Yokohama City University Medical Center), Kazutaka Koganei (Inflammatory Bowel Disease Center, Yokohama Municipal Citizen’s Hospital), Kiyonori Kobayashi (Research and Development Center for New Medical Frontiers, Kitasato University School of Medicine), Kenji Kobayashi (Division of Gastroenterology, Kameda Kyobashi Clinic), Masayuki Saruta (Division of Gastroenterology and Hepatology, Department of Internal Medicine, The Jikei University School of Medicine), Toshiaki Tanaka (Department of Surgical Oncology, University of Tokyo), Hiroshi Nakase (Department of Gastroenterology and Hepatology, Sapporo Medical University School of Medicine), Masakazu Nagahori (Department of Gastroenterology and Hepatology, Tokyo Medical and Dental University), Fumihito Hirai (Inflammatory Bowel Disease Center, Fukuoka University Chikushi Hospital), Satoshi Motoya (IBD center, Sapporo Kosei Hospital).

### Evaluation Committee

Chair: Toshiyuki Matsui (Department of Gastroenterology, Fukuoka University, Chikushi hospital).

Vice-Chair: Takanori Kanai (Department of Gastroenterology and Hepatology, Keio University Hospital).

Members: Kenichi Takahashi (Department of Colorectal Surgery, Tohoku Rosai Hospital) Yoshinori Noguchi (Division of General Internal Medicine, Japanese Red Cross Nagoya Daini Hospital) Kenji Watanabe (Department of Gastroenterology, Osaka City General Hospital).

### Observer

Yasuo Suzuki (Department of Internal Medicine, Toho University Sakura Medical Center).
